# PRL-3 facilitates Hepatocellular Carcinoma progression by co-amplifying with and activating FAK

**DOI:** 10.7150/thno.42069

**Published:** 2020-08-18

**Authors:** Qiming Zhou, Qianlei Zhou, Qinghua Liu, Zhanghai He, Yongcong Yan, Jianhong Lin, Zheng Chen, Chuanchao He, Kai Mao, Jie Wang, Zhenyu Zhou, Zhiyu Xiao, Jianlong Zhang

**Affiliations:** 1 Department of Hepatobiliary Surgery, Sun Yat-Sen Memorial Hospital, Sun Yat-Sen University, Guangzhou 510120, China.; 2 Guangdong Province Key laboratory of Malignant Tumour Epigenetics and Gene Regulation, Sun Yat-Sen Memorial Hospital, Sun Yat-Sen University, Guangzhou 510120, China.; 3 Department of Thyroid Surgery, Sun Yat-Sen Memorial Hospital, Sun Yat-Sen University, Guangzhou, 510120, China.; 4 Department of Pathology, Sun Yat-Sen Memorial Hospital, Sun Yat-Sen University, Guangzhou 510120, China.

**Keywords:** HCC, PRL-3, Copy number variations, Phosphatase, TGFB1

## Abstract

**Background:** In addition to protein tyrosine kinases, accumulating evidence has shown that protein tyrosine phosphatases (PTPs) are suitable therapeutic targets in cancer. PRL-3 is a PTP member that has been well studied in many malignant tumours. The goal of the present study was to elucidate the role of PRL-3 in hepatocellular carcinoma (HCC), which remains largely unknown.

**Methods:** Bioinformatic and immunohistochemical analyses were performed to analyse PRL-3 expression in HCC tissue samples and determine its clinical relevance. PRL-3 gene copy number variations were evaluated by bioinformatic analysis and quantitative-genomic polymerase chain reaction. The biological functions of PRL-3 were investigated *in vivo* and vitro. Gene microarray assays, RT-qPCR, western blotting and luciferase experiments were performed to identify the downstream effectors of PRL-3 that mediate its functions in HCC.

**Results:** PRL-3 expression was upregulated in HCC samples from public databases and in cohort samples from our centre. High PRL-3 expression was associated with poor prognosis. Copy number gains and amplification of chromosome 8q24.3 in HCC were determined to be positively correlated with the PRL-3 overexpression. PRL-3 overexpression promoted HCC cell proliferation, migration and adhesion, while its loss had the opposite effects. Further study showed that focal adhesion kinase (FAK) was co-amplified and co-expressed with PRL-3 in HCC. Interestingly, PRL-3 also promoted the phosphorylation of FAK, which subsequently mediated the oncogenic functions of PRL-3 in HCC cells. Moreover, TGFB1 was identified as a downstream molecule of PRL-3. TGF-β signalling was shown to mediate the PRL-3-induced activation of FAK. Furthermore, the p38 and PI3K/AKT pathways were observed to mediate the PRL-3-induced expression of TGFB1 and the subsequent activation of FAK, while the activation of FAK in turn stimulated activation of the p38 and PI3K/AKT pathways, forming a PRL-3-triggered AKT/p38/TGFB1/FAK positive feedback loop.

**Conclusion:** Collectively, our findings indicate that the PTP PRL-3 plays a crucial role in the progression of HCC and provides an example of how co-amplified genes work together in HCC.

## Introduction

Protein tyrosine kinases (PTKs) are a group of phosphorylation-related enzymes that are frequently dysregulated in malignant tumours, including hepatocellular carcinoma (HCC), and a number of PTKs have served as therapeutic targets for cancer 1. However, it should be noticed that protein tyrosine phosphatases (PTPs), another group of phosphorylation-related enzymes, also play an important role on tumour initiation and progression 2. Although accumulating evidence has shown that PTPs are suitable therapeutic targets in cancer, compared to PTKs, relatively little is known regarding the role of PTPs in HCC.

The protein tyrosine phosphatase 4A (PTP4A) family, commonly known as phosphatases of regenerating liver (PRLs), is a three-member family of phosphatases that play crucial oncogenic roles in a variety of human cancers 3. The inhibition of PRLs shows promise for cancer treatment, and several PRLs inhibitors have been reported 3. Therefore, it is important to elucidate the roles of PRLs in HCC. The results of our previous study showed that PRL-1 is overexpressed in HCC and promotes HCC cell migration and invasion through endothelial-mesenchymal transition (EMT) induction 4. In addition to PRL-1, PRL-3 has also been reported to be upregulated in HCC and have a negative impact on the prognosis of HCC patients 5,6. However, a detailed understanding of the molecular mechanism by which PRL-3 promotes HCC is still lacking.

Copy number variations (CNVs) occur early in hepatocarcinogenesis and remain relatively stable throughout tumour progression 7. CNVs include gene deletions, copy number gains and amplification events, all of which are frequently observed in HCC and lead to the dysregulation of numerous tumour suppressor genes and driver oncogenes 8. Copy number gains and the amplification of specific chromosomal loci not only affect individual genes but rather results in the overexpression of neighbouring genes 8. Some of the overexpressed neighbouring genes may cooperate to promote tumour initiation and progression, but the associated mechanism depends on the molecular relationship between the neighbouring genes themselves 9,10. Understanding the mechanism which such co-amplified genes cooperate may help to identify new therapeutic strategies for HCC.

In the present study, we showed that the PRL-3 gene (PTP4A3) is amplified in HCC. We also observed that FAK, another oncogene located adjacent to the PRL-3 gene, is co-amplified with the PRL-3 gene in HCC. Furthermore, we provide evidence that PRL-3 overexpression dose not regulate FAK expression but rather promotes FAK phosphorylation through the TGFB1/Src signalling pathway. Phosphorylated FAK mediates the pro-migration and pro-adhesion effect of PRL-3. These findings further elucidate the oncogenic role of PRL-3 in HCC and provide insights into how the co-amplification of neighbouring genes functions to promote cancer.

## Methods and Materials

### Patients and clinical samples

A tissue microarray comprising 96 pairs of primary HCC tissues together with their clinical and prognosis data were acquired from the specimen library of the Department of Hepatobiliary Surgery, Sun Yat-Sen Memorial Hospital (SYSMH), Sun Yat-Sen University (Guangzhou, China). The samples were obtained from HCC patients who had undergone hepatic resection between March 1, 2015 and February 1, 2016. These samples were stored in liquid nitrogen for further nucleic acid extraction and paraffin embedding. All patients and their corresponding tissue samples had been confirmed by histopathologists. None of the patients underwent preoperative chemotherapy or interventional radiology. The study protocol was approved by the Ethics Committee of Sun Yat-Sen Memorial Hospital (SYSMH), and informed consent was obtained from each patient. Detailed patient clinical data is listed in Table S1. Cancer clinical staging was performed according to the AJCC/UICC tumour-node-metastasis (TNM) stage (2010).

### Immunohistochemistry (IHC)

IHC was performed as described previously 11, and scoring of the tissue microarray was independently completed by two pathologists who had no knowledge of the patients' clinical data. The staining intensity was scored as follows: 0 (Negative); 1 (Light brown); 2 (Brown); and 3 (Dark brown). The proportion of positive-stained cells was scored as follows: 0-10%, 0; 11-25%, 1; 26-50%, 2; 51-75%, 3; and 75-100%, 4. IHC staining total scores were obtained by multiplying the intensity score by the proportion score. We defined high expression as a total score of greater than or equal to 6 points, with other scores defined it low expression. The antibody against PRL-3 was diluted 1:100 (ab50276, Abcam, Cambridge, UK). Other antibodies used for IHC staining included those against the proteins FAK (1:150, #71433, Cell Signaling Technology, MA, USA), p-FAK (1:100, YP0739, ImmunoWay, USA), and TGFB1(1:200, YT4632, ImmunoWay, USA).

### DNA purification and quantitative-genomic PCR

Genomic DNA from HCC tissues and the corresponding adjacent liver tissues was extracted using a QIAamp DNA FFPE Kit (QIAGEN Sciences, Hilden). Quantitative-genomic polymerase chain reaction (Q-gPCR) was performed to quantify genomic amplification of PRL-3 in triplicate samples using iQ™ Supermix and the iCycler iQ™ Real‑Time PCR Detection system (both from Bio‑Rad Laboratories, Hercules, CA, USA). To normalize PRL-3 gene copy number per cell, β-actin was used as an endogenous reference, as previously reported 12. The ΔCt values were calculated as Ct (PRL-3)-Ct (β-actin) for each sample, and the copy number relative to the corresponding liver tissues was determined as 2^-ΔΔCt^, where ΔΔCt = ΔCt(tumour)-ΔCt (corresponding normal tissue) 13. The DNA ratios for the tumour tissues relative to the corresponding normal tissues that were equal to or greater than 2-fold were defined as positive genomic gains.

### Cell-matrix adhesion assay

To assess cell-matrix adhesion, 96-well plates were coated with 10 μg/mL of fibronectin in PBS at 37°C overnight, after which the plates were blocked with 1% BSA for 1 h at 37°C and then washed twice with PBS. Subsequently, 10^4^ cells in 100 µL was added to each coated well, and the cells were allowed to adhere at 37°C for 30 min. After the unattached cells were gently washed away with PBS, CCK-8 solution was added to each well at a final concentration of 10% and incubated for 2 h. Subsequently, the absorbance of the samples was measured at 450 nm using a microplate reader, with the change in adhesion ability reported as the ratio of the absorbances among different groups.

### Tumourigenesis and metastasis analyses in nude mice

Female BALB/c nude mice (4-6 weeks of age, 16-20 g) were purchased from the Laboratory Animal Center of Sun Yat-Sen University. For the tumourgenesis assay, the nude mice were subcutaneously injected with HCC cells near the right scapula with stable cell lines (5 × 10^6^ in 200 μL of PBS). The nude mice were sacrificed 4 weeks after injection. The tumours from the nude mice were dissected and measured with calliper, and the tumour volume was calculated according to the formula Volume = Length × Width^2^ × 0.5. For pulmonary metastasis assays, the nude mice were injected through the lateral tail veins with the HCC cell lines (2 × 10^6^ in 100 μL of PBS). The mice were euthanized 6 weeks after injection, and the lungs were collected, fixed in 4% paraformaldehyde and embedded in paraffin. Serial sections of mouse lungs were stained with H&E, and the metastatic status was confirmed by the observation of the sections under a light microscope. All experimental procedures involving animals were in performed accordance with the National Institutes of Health Guide for the Care and Use of Laboratory Animals and approved by the Animal Ethical and Welfare Committee of SYSU.

### Dual-luciferase reporter assay

The full length TGFB1 promoter regions, sequence 1 (0 to -500 bp region of TGFB1 promoter) and sequence 2 (-1500 to -2000 bp region of TGFB1 promoter) were cloned into the vector pGL4.18[luc2P/Neo] (Promega Corp., Fitchburg, WI, USA). An AP-1 reporter vector (pGMAP-1 Lu) with the AP-1 response element was purchased from Genomeditech, LTD (Shanghai, China). Cells were cotransfected with the indicated plasmids together with the reporter plasmid and the pGL4.74[hRluc/TK] vector using the liposome method. Cells were harvested 24 h later, and then firefly and Renilla luciferase activities were assayed with dual-luciferase assay system (Promega Corp., Fitchburg, WI, USA) according to the manufacturer's instructions. Firefly luciferase activity was normalized to Renilla luciferase activity. All experiments were, performed in triplicate, and the luminescence values for each group were statistically analysed.

### Statistical analysis

The comparison of quantitative data between two groups was performed by using Student's *t*-test, while ANOVA was used for comparisons of quantitative data for multiple groups. The Kaplan-Meier method and log-rank test were used to generate and compare survival curves. The association between PRL-3 expression and the clinicopathological features were analysed by χ2 test. The Pearson correlation analysis was performed to assess the relationships between PRL-3 CNVs and mRNA levels, PRL-3 and FAK in CNVs levels, PRL-3 and FAK in mRNA levels, and PRL-3 and TGFB1 mRNA levels. The χ^2^ test was used to analyse the correlation between the protein expression levels of PRL-3 and FAK as well as between PRL-3 and TGFB1 in HCC tissues. Statistical analyses and figure generation were performed using Prism 6.0 (GraphPad Software, Inc., La Jolla, CA, USA) and SPSS version 25.0 (IBM). Differences where *P* value < 0.05 were considered to be significant.

The detailed descriptions of other experiments involved in the article were presented in the Supplementary Methods and Materials.

## Results

### PRL-3 is overexpressed and associated with poor prognosis in HCC

**Figure 1:**
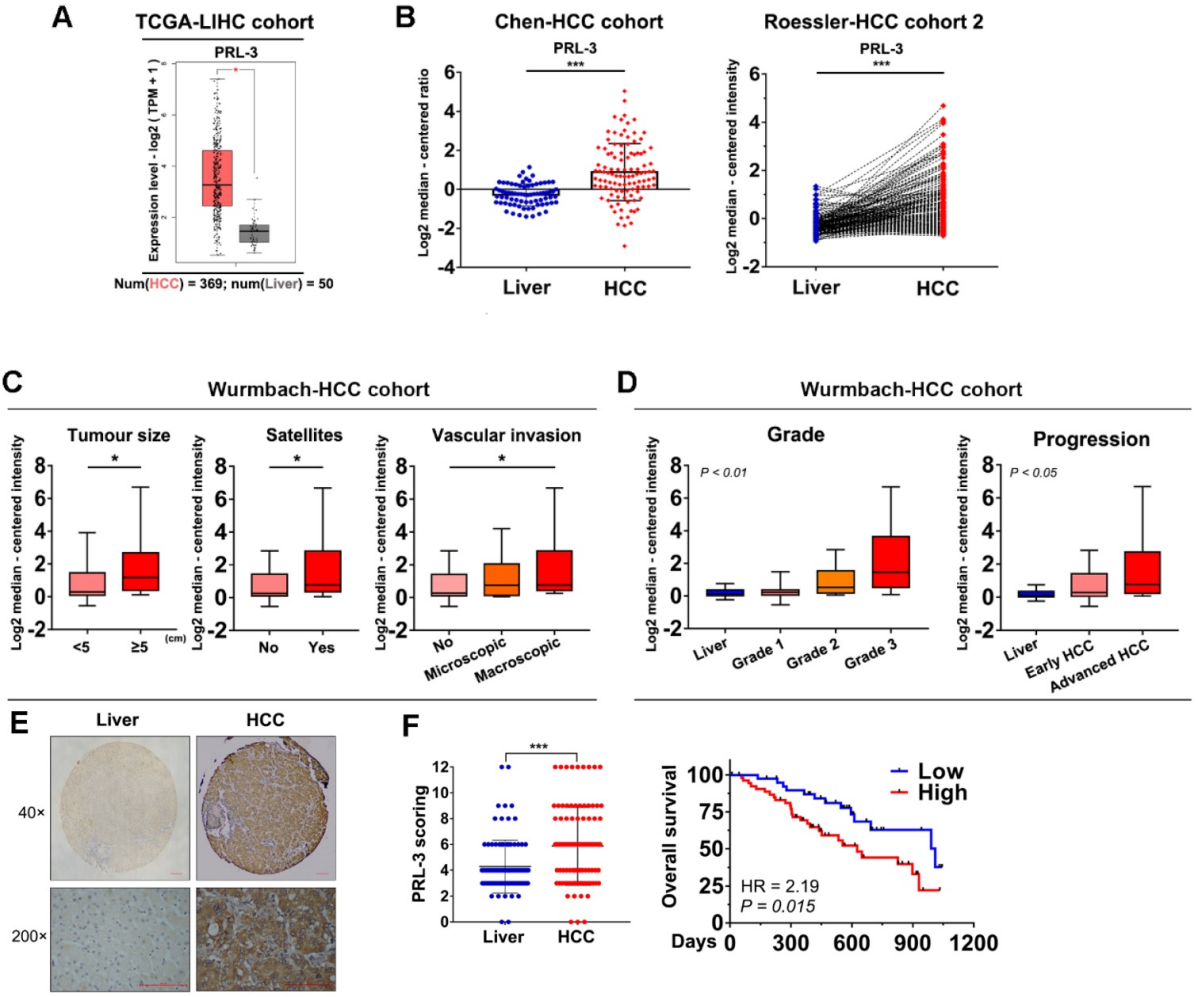
**Expression level and prognosis value of PRL-3 in HCC. (A)** Bioinformatic analysis of the mRNA expression level of PRL-3 in HCC and normal liver tissues. The data were obtained from the GEPIA database (http://gepia.cancer-pku.cn/index.html). **(B)** Bioinformatic analysis of the mRNA expression level of PRL-3 in the Chen-HCC cohort and the Roessler-HCC cohort 2. The data were obtained from the ONCOMINE database (www.Oncomine.Org).** (C)** Bioinformatic analysis of the mRNA expression level of PRL-3 in HCC patients grouped by tumour size, presence of satellites and vascular invasion. The data were derived from the Wurmbach-HCC cohort in the ONCOMINE database (www.Oncomine.Org). **(D)** Bioinformatic analysis of the mRNA expression level of PRL-3 in the HCC patients grouped by tumour grade and progression. One-way ANOVA test. The data were derived from the Wurmbach-HCC cohort in ONCOMINE database (www.Oncomine.Org). **(E)** Representative images of IHC staining with anti-PRL-3 in 96 paired HCC and liver tissues (40×, 200×, magnification, scale bars: 100 µm) and a dot chart of the IHC scoring. The results are presented as the means ±SD.** (F)** Kaplan-Meier overall survival curve of the two HCC groups: High PRL-3 expression (red) and low PRL-3 expression (blue). *, *P* < 0.05; **, *P* < 0.01; ***, *P* <0.001.

We used the GEPIA database to preliminarily assess the level of PRL-3 expression in HCC and normal liver tissues, and the results showed a significantly higher expression of PRL-3 in HCC tissues (Figure 1A). Consistently, higher PRL-3 expression was observed in HCC tissues from other independent cohorts in the ONCOMINE database (Figure 1B; Figure S1A). We then performed a bioinformatic analysis to assess the level of PRL-3 expression in different subgroups. In the Wurmbach-HCC cohort, higher PRL-3 expression levels were observed in HCC patients with large tumour sizes, the coexistence of satellite lesions and the presence of vascular invasion (Figure 1C). In the Liao-HCC cohort, PRL-3 expression was significantly higher in HCC intrahepatic metastatic lesions than in primary lesions (Figure S1B). Furthermore, both the Wurmbach-HCC and TCGA-LIHC cohorts showed that as the tumour grade and status increased, there was an increased tendency for PRL-3 expression (Figure 1D; Figure S1C). Kaplan-Meier analysis using TCGA-LIHC data revealed that patients with higher PRL-3 expression exhibited a poorer overall survival (Figure S1D). The results of these bioinformatic analyses using public databases suggested that PRL-3 may be an important oncogene in HCC. We further validated these results using data from our centre. PRL-3 expression was detected by IHC in a tissue microarray comprising 96 pairs of HCC and adjacent liver tissues. The results indicated that PRL-3 was upregulated in HCC tissues compared with adjacent liver tissues (Figure 1E). High PRL-3 expression was positively correlated with a larger tumour size, vascular invasion and advanced TNM stage (Table S1). Furthermore, HCC patients with high PRL-3 expression had a shorter overall survival time compared with that observed in cases with low PRL-3 expression (Figure 1F). Thus, the data from both public databases and our centre indicate that PRL-3 is an important oncogene in HCC.

### Genomic gains and amplification of PRL-3 gene are associated with its overexpression in HCC

**Figure 2:**
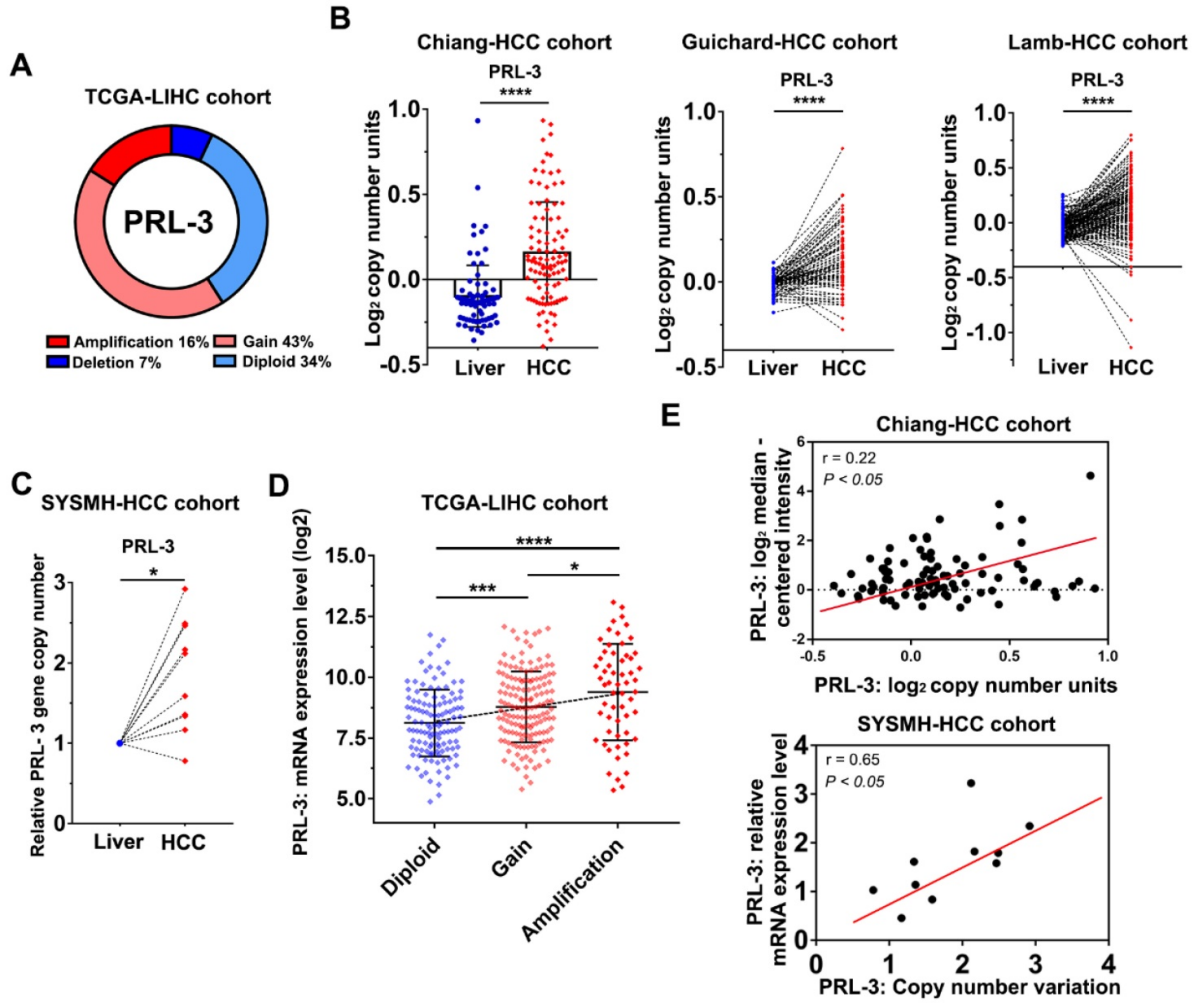
** Copy number gains and amplification are responsible for PRL-3 overexpression in HCC. (A)** The copy number variations of PRL-3 in the TCGA-LIHC cohort. The data were obtained from the cBioPortal database (http://www.cbioportal.org/). **(B)** The increased PRL-3 gene copy number in the Chiang-HCC, Guichard-HCC and Lamb-HCC cohorts. The data were obtained from the ONCOMINE database (http://www.oncomine.org/). **(C)** Results of quantitative-genomic PCR for detecting PRL-3 gene copy number variations in 10 paired HCC and liver tissues from our centre (Sun Yat-Sen Memorial Hospital, SYSMH). **(D)** A plot showing the relationship between copy number status and mRNA expression of the PRL-3 gene in the TCGA-LIHC cohort. Diploid, two alleles present; Gain, low-level gene amplification event; Amplification, high-level gene amplification event. **(E)** Pearson correlation analysis between the PRL-3 gene copy number and its mRNA expression in the Chiang-HCC cohort from the ONCOMINE database and in our 10 HCC tissue samples. *, *P* < 0.05; **,* P* < 0.01; ***, *P* <0.001, ****,* P* < 0.0001.

To investigate the oncogenic role of PRL-3 in HCC, we first evaluated how PRL-3 was upregulated in HCC. We noticed that the PRL-3 gene (PTP4A3) is located on human chromosome 8q24.3, a region that frequently exhibits genomic gain and amplification in HCC 14. Thus, we speculated that the high expression status of PRL-3 in HCC may be attributed to CNV. As expected, copy number gains and gene amplification of the PRL-3 gene occurred in 43% (158/364) and 16% (59/364) of HCC tissues, respectively, in the TCGA-LIHC dataset (Figure 2A). HCC cohorts in the ONCOMINE database with copy number information also showed a significantly higher PRL-3 gene copy number in HCC than in liver tissues (Figure 2B). Moreover, we assessed the PRL-3 gene copy number in 10 pairs of HCC and adjacent liver tissues from our centre and again observed an increased PRL-3 gene copy number in HCC tissues (Figure 2C). We next confirmed whether the high PRL-3 gene copy number was positively correlated with its increased expression. Data from the TCGA-LIHC and Chiang-HCC cohorts and HCC samples from our centre all consistently indicated a positive correlation between the copy number and mRNA expression of the PRL-3 gene (Figure 2D, E). Taken together, these data indicate that PRL-3 gene amplification correlates with increased PRL-3 expression in HCC.

### PRL-3 is involved in the proliferation and metastasis of HCC cells *in vitro* and *in vivo*

**Figure 3:**
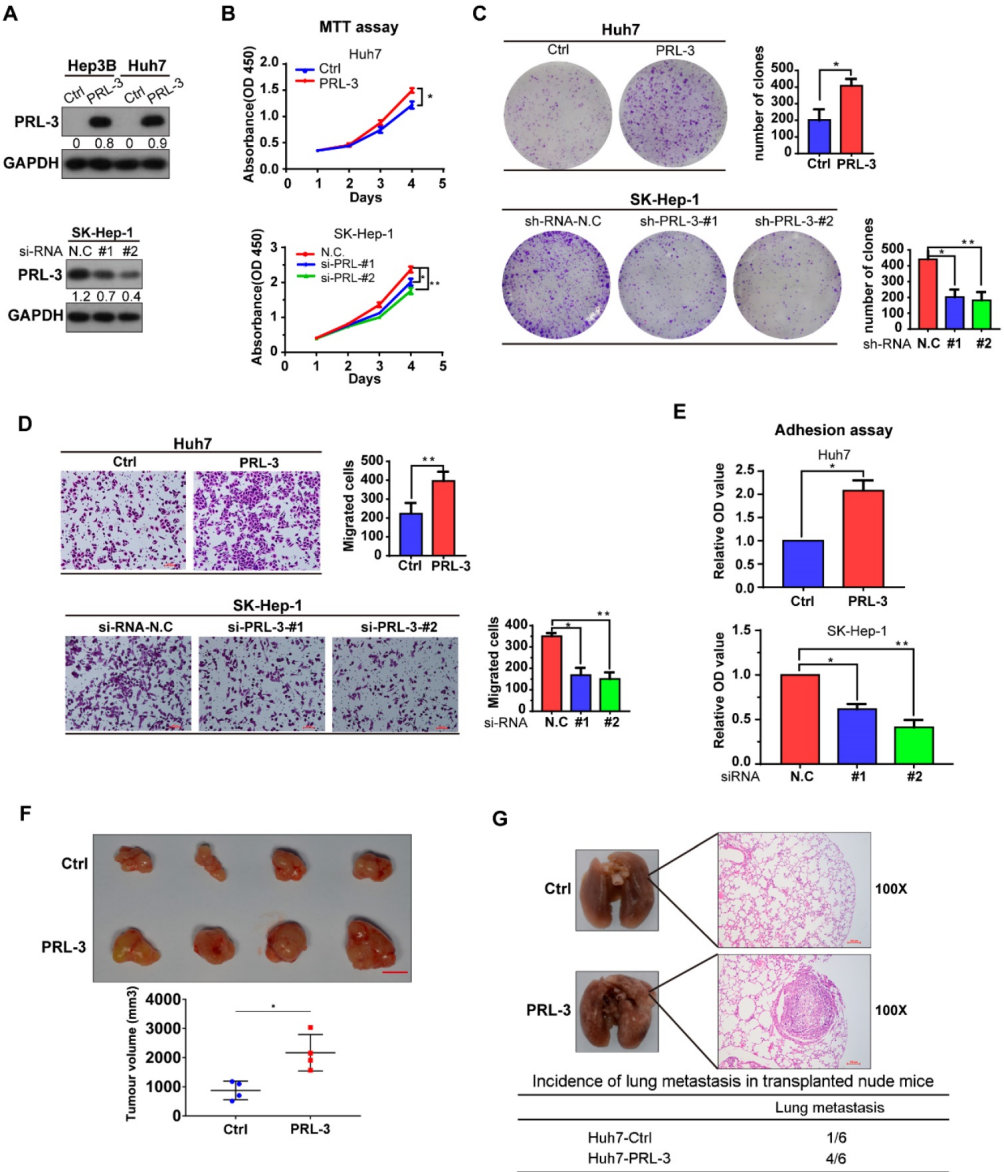
** Effect of PRL-3 expression on HCC cell proliferation and metastasis *in vitro* and vivo. (A)** PRL-3 overexpression in Hep3B and Huh7 cells and PRL-3 knockdown in SK-Hep-1 cells was confirmed by western blot. **(B, C, D)** MTT assay **(B)**, clone formation assay **(C)** and Transwell assay **(D)** of HCC cells with PRL-3 overexpression or knockdown. **(E)** Cell adhesion in HCC cells with PRL-3 overexpression or knockdown was measured by a cell-matrix adhesion assay. **(A-D)** The results are presented as the means ± standard error of the mean of three independent experiments. *, *P* < 0.05, **, *P* < 0.01. **(F)** Tumours derived from nude mice subcutaneously implanted with Huh7-Ctrl or Huh7-PRL-3 cells. Scale bar: 1 cm. Tumour volumes are presented as the means ± SEM. **(G)** Lungs derived from nude mice with tail intravenous injection of Huh7-Ctrl or Huh7-PRL-3 cells and the corresponding H&E staining images. Scale bar: 100 µm. *, *P* < 0.05.

As we observed that PRL-3 was highly expressed in HCC tissues and associated with the poor prognosis of HCC patients, we assessed the oncogenic function of PRL-3 in HCC. The endogenous expression of PRL-3 in HCC cell lines and immortalized hepatocyte LO2 cells was evaluated (Figure S2A). Based on the differences in PRL-3 expression observed in the HCC cell lines, we chose Huh7 and Hep3B to establish cell lines stably overexpressing PRL-3, while the cell lines SK-Hep-1 and HepG2 were used for PRL-3 knockdown experiments (Figure 3A; Figure S2B). We observed that PRL-3 overexpression significantly enhanced the proliferation and migration of Huh7 cells, whereas of PRL-3 knockdown markedly reduced SK-Hep-1 and HepG2 cell proliferation and migration (Figure 3B-D; Figure S2C-E). Because PRL-3 has been demonstrated to regulate intracellular pathways involved in focal adhesion 15, we tested whether PRL-3 altered the adhesion ability of HCC cells to extracellular matrix (ECM) substrates. Cell-matrix adhesion assay results showed that PRL-3 overexpression led to a significant increase in cell adhesion to fibronectin, whereas this adhesion was inhibited by PRL-3 knockdown (Figure 3E; Figure S2F). Furthermore, subcutaneous xenograft model and *in vivo* lung metastasis assay results indicated that PRL-3 overexpression significantly enhanced xenografted tumour growth and increased the incidence of lung metastasis (Figure 3F, G), while the PRL-3 knockdown cell line exhibited the opposite effects (Figure S2G and S2H). These results suggest that PRL-3 upregulation in HCC may exert its oncogenic function by regulating HCC cells with respect to their proliferation, migration and adhesion to ECM substrates.

### FAK is co-amplified with PRL-3

**Figure 4:**
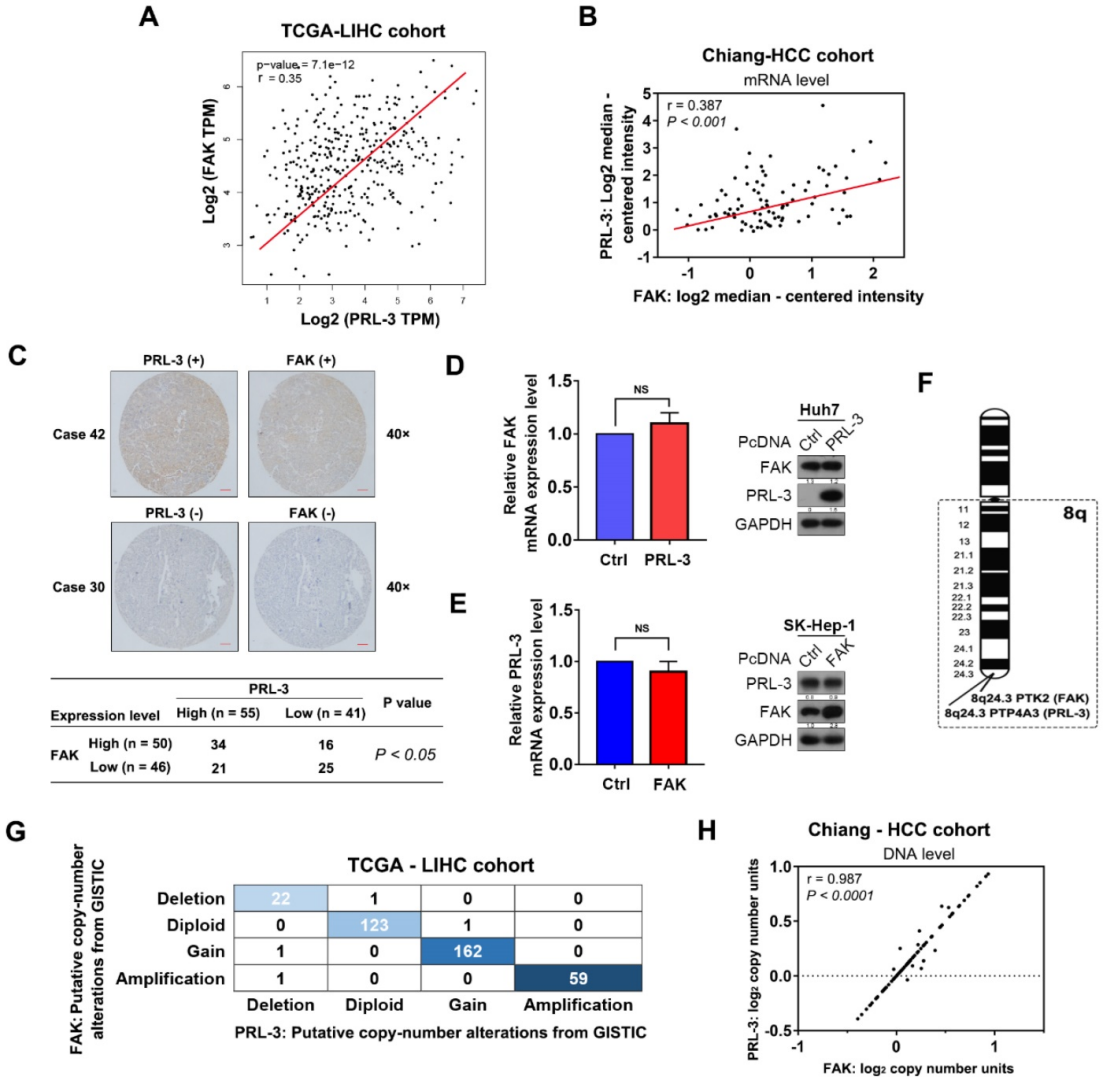
** FAK is co-amplified with PRL-3. (A)** Positive correlation between PRL-3 and FAK mRNA expression levels in the TCGA-LIHC cohort. The data was derived from the GEPIA database (http://gepia.cancer-pku.cn/index.html). **(B)** Positive correlation between PRL-3 and FAK mRNA expression levels in the Chiang-HCC cohort. The data were obtained from the ONCOMINE database (http://www.oncomine.org). **(C)** Positive correlation between PRL-3 and FAK protein expression levels in the 96 HCC tissue samples. Pearson χ^2^ test, *P* < 0.05. **(D and E)** No regulatory relationships were observed between PRL-3 and FAK at the mRNA and protein levels.** (F)** Position of the PTP4A3 gene (encoded PRL-3) and PTK2 gene (encoded FAK) on chromosome 8q based on the NCBI database (http://www.ncbi.nlm.nih.gov/). **(G)** Positive correlation in copy number variations between the PRL-3 and FAK genes in 370 HCC samples from the TCGA-LIHC cohort. The data were obtained from the cBioPortal database (http://www.cbioportal.org/). **(H)** Positive correlation in copy number variations between the PRL-3 and FAK genes in the Chiang-HCC cohort. The data were obtained from the ONCOMINE database (http://www.oncomine.org). The results are presented as the means ± standard error of the mean of three independent experiments. NS, not significant.

Given the findings described above, we next investigated how PRL-3 promotes HCC cell adhesion to ECM, a key regulator of multiple cellular processes in cancer cells 16. Focal adhesion kinase (FAK) plays an important role in regulating cell focal adhesion and has been shown to promote cell proliferation, migration and adhesion in HCC 17,18. Coincidentally, the TCGA-LIHC dataset, Chiang-HCC cohort, Jia-HCC cohort, Roessler-HCC cohort 2 and Roessler-HCC cohort 1 from the ONCOMINE database all showed a positive correlation between PRL-3 and FAK expression at the mRNA level (Figure 4A, B; Figure S3A). Subsequently, a positive relationship between PRL-3 and FAK protein levels was further confirmed in our tissue microarray using IHC (Figure 4C). However, neither PRL-3 nor FAK overexpression affected the mRNA or protein expression of each other (Figure 4D, E), suggesting that the highly positive relationship between PRL-3 and FAK expression may not result from the direct regulation on mRNA or protein levels mediated by each other. Interestingly, the FAK gene (PTK2) is also located on human chromosome 8q24.3, adjacent to the PRL-3 gene (Figure 4F). Therefore, we assessed whether CNV was responsible for the observed co-expression of PRL-3 and FAK. Interestingly, data from TCGA-LIHC dataset, Chiang-HCC cohort, Lamb-HCC cohort, Guichard-HCC cohort 1 and Guichard-HCC cohort 2 all showed a significant and positive relationship between PRL-3 and FAK on CNV status (Figure 4G, H; Figure S3B). Taken together, these data indicate the co-amplification of PRL-3 and FAK in HCC, which results in the co-expression of the two genes.

### PRL-3 functions in HCC by phosphorylating FAK

**Figure 5:**
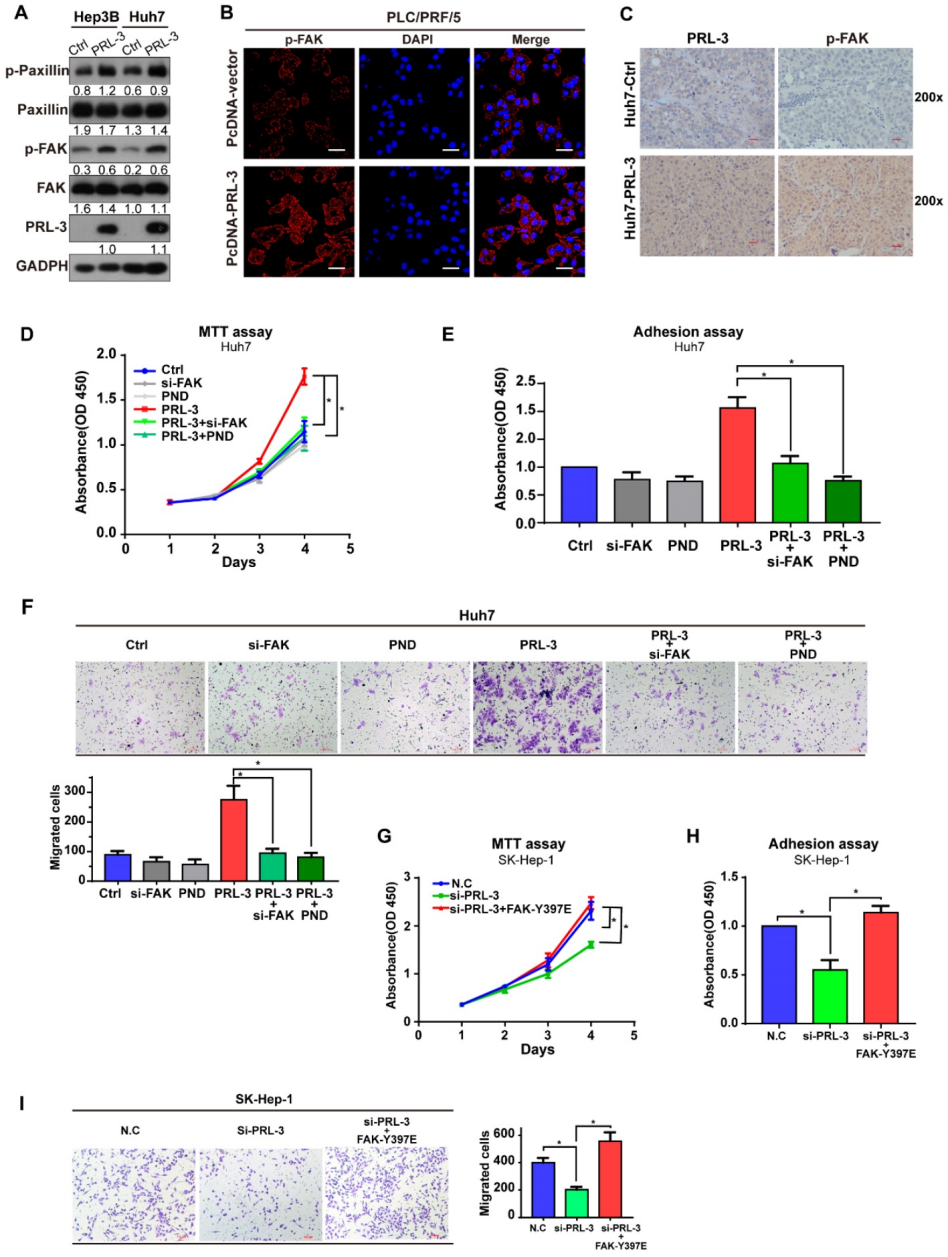
** PRL-3 functions by phosphorylating FAK. (A)** Western blot showing the role of PRL-3 on the phosphorylation of FAK and paxillin. **(B)** Phosphorylation of FAK by PRL-3 was further confirmed by immunofluorescence staining. Scale bar: 50 µm. **(C)** The expression of PRL-3 and phosphorylated FAK in subcutaneous xenograft tumours of mice was assessed by IHC staining. Scale bar: 10 µm. **(D, E, F)** The effect of FAK knockdown or inhibition on Huh7 cells with PRL-3 overexpression was evaluated by MTT **(D),** cell adhesion assays **(E)** and Transwell (**F**). PND-1186 was used to inhibit FAK activity at a concentration of 0.1 µM for 24 h. (**G, H, I**) The effect of a constitutively active FAK mutant (FAK-Y397E) on SK-Hep-1 cells with PRL-3 knockdown were evaluated by MTT **(G),** cell adhesion assays (**H**) and Transwell **(I)**. The data are presented as the mean and SD of three independent experiments. *, *P* < 0.05; **, *P* < 0.01.

Although our data showed that PRL-3 and FAK did not regulate the expression of each other, the colocalization on chromosome and co-amplification of both genes indicated the potential existence of a functional relationship between them. We observed that PRL-3 overexpression promoted the phosphorylation of FAK and its downstream signalling target, paxillin (Figure 5A), whereas PRL-3 knockdown inhibited the phosphorylation of FAK (Figure S4A). The enhanced phosphorylation of FAK by PRL-3 was further confirmed by immunofluorescence staining results (Figure 5B). In addition, the positive correlation between PRL-3 and phosphorylated FAK was also demonstrated in analyses of subcutaneous xenograft and lung metastasis tumours of mice (Figure 5C; Figure S4B). Furthermore, assays were performed in which siRNA was used to knockdown the expression of FAK and the inhibitor PND-1186 was used to prevent the activation of FAK (Figure S4C). Both knockdown of FAK expression and inhibition of FAK activation attenuated the effect of PRL-3 on HCC cell proliferation, adhesion and migration (Figure 5D-F; Figure S4D-F). In contrast, overexpression of a constitutively active FAK mutant (FAK-Y397E) rescued the effect of PRL-3 knockdown on these phenotypes (Figure S4G; Figure 5G-I). These results suggest that PRL-3 promotes HCC cell proliferation, migration and adhesion through activation of FAK.

### PRL-3 induces FAK activation through TGFB1/Src in HCC

**Figure 6:**
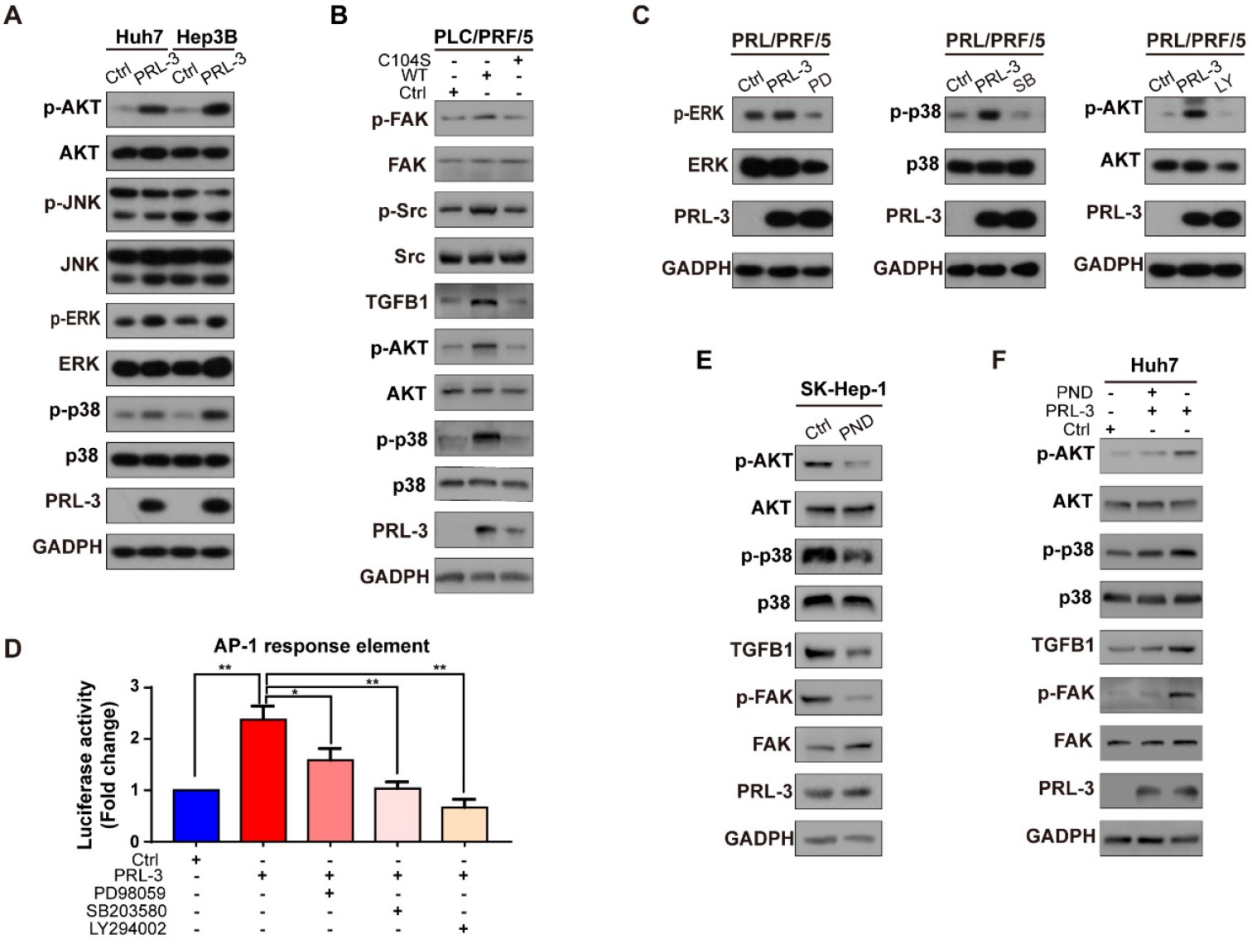
** PRL-3 induces FAK activation through TGFB1/Src in HCC. (A)** Positive correlation between PRL-3 and TGFB1 mRNA expression levels in the Woo-HCC cohort, Hoshida-HCC cohort, Chiang-HCC cohort and Roessler-HCC cohort 2. The data were obtained from the ONCOMINE database (www.oncomine.org). **(B and C)** RT-qPCR (**B**) and western blot (**C**) assays were performed to assess PRL-3 regulation of TGFB1 mRNA and protein expression in PRL-3-overexpressing Huh7 and Hep3B cells as well as PRL-3 knockdown SK-Hep-1 cells. **(D)** Secreted TGF-β levels in the supernatants of PRL-3-overexpressing Huh7 and Hep3B cells as well as PRL-3 knockdown SK-Hep-1 cells, as determined by ELISA.** (E)** Positive correlation between PRL-3 and FAK expression in 96 HCC tissue samples. Pearson χ^2^ test, *P* < 0.01. **(F-I)** Western blot was performed to assess the expression of molecules related to the TGFB1/Src/FAK pathway in different HCC cell lines. (**F**) Huh7 cells stably overexpressing PRL-3 were treated with the TGF-β receptor inhibitor SB-431542 (1 μM) for 24 h. (**G**) PRL-3-silenced SK-Hep-1 cells were treated with human recombinant TGFB1 (2 ng/mL) for 24 h**. (H)** Huh7 cells stably overexpressing PRL-3 were treated with Src siRNA. **(I)** PRL-3-silenced SK-Hep-1 cells overexpressing a constitutively active Src mutant (Src-Y527F). The results are presented as the means ± standard error of the mean of three independent experiments. *, *P* < 0.05, **, *P* < 0.01.

To elucidate the mechanism underlying the PRL-3-induced activation of FAK, we first compared the gene expression profiles between Huh7-PRL-3 and Huh7-Ctrl cells through microarray analysis. The results revealed 59 upregulated and 49 downregulated genes from the three independent microarray assays (Figure S5A). Next, we compared these dysregulated genes with those that were significantly correlated with PRL-3 expression in the TCGA-LIHC dataset, resulting in the identification of 4 upregulated genes and 1 downregulated gene (Figure S5A). We selected TGFB1 for further study, as it has been reported to promote FAK activation 19, 20. The positive correlation between PRL-3 and TGFB1 on mRNA level was also observed in four other HCC datasets from ONCOMINE (Figure 6A). The regulation of TGFB1 by PRL-3 was confirmed by RT- PCR and western blot analyses (Figure 6B and 6C; Figure S5B). Moreover, ELISA results showed that PRL-3 overexpression caused an increase in the secretion of TGF-β by HCC cells, while its loss had the opposite effects (Figure 6D). A positive correlation between PRL-3 and TGFB1 was also observed in our tissue microarray results (Figure 6E). In addition, we also noted that Src, which had been demonstrated to mediate TGFB1-induced FAK activation 21, was also regulated by PRL-3 (Figure 6C), suggesting a PRL-3-TGF-β-Src signalling occurs in HCC cells. To determine whether PRL-3 promotes FAK activation through TGF-β-Src signalling in HCC, we assessed the effects of a TGF-β receptor inhibitor, SB431542, and human recombinant TGF-β on FAK and Src activation. SB431542 treatment significantly attenuated the PRL-3-dependent activation of FAK and Src (Figure 6F), while TGF-β treatment notably promoted FAK and Src activation in PRL-3 knockdown cells (Figure 6G). We then explored whether Src is also involved in this regulation, and our results showed that Src knockdown decreased the level of FAK phosphorylation in PRL-3-overexpressing cells, without affecting TGFB1 expression (Figure 6H). In contrast, overexpression of a constitutively active Src mutant (Src-Y527F) could rescue the downregulation of FAK phosphorylation in PRL-3 knockdown cell lines (Figure 6I). These data indicate that PRL-3 induces FAK activation by enhancing TGFB1 expression and promoting downstream Src signalling in HCC.

Integrins are another well-documented activator of FAK 22, 23. Through data mining using a TCGA-LIHC dataset, we observed that ITGB1 (integrin β1) has a significant correlation with FAK, PRL-3, TGFB1 and TGFBR1 in HCC (Figure S5C). Further study showed that integrin β1 and TGFBR1 colocalized in the cell membrane (Figure S5D), while knockdown of integrin β1 inhibited the PRL-3-induced activation of FAK (Figure S5E). These additional data indicate there may be a crosstalk between TGF-β/TGFBR1 and integrin β1 in mediating the PRL-3-induced activation of FAK, which further indicate the important role of TGFB1 as a downstream of PRL-3 in HCC.

### PRL-3 enhances TGFB1 transcription through the AP-1 complex

**Figure 7:**
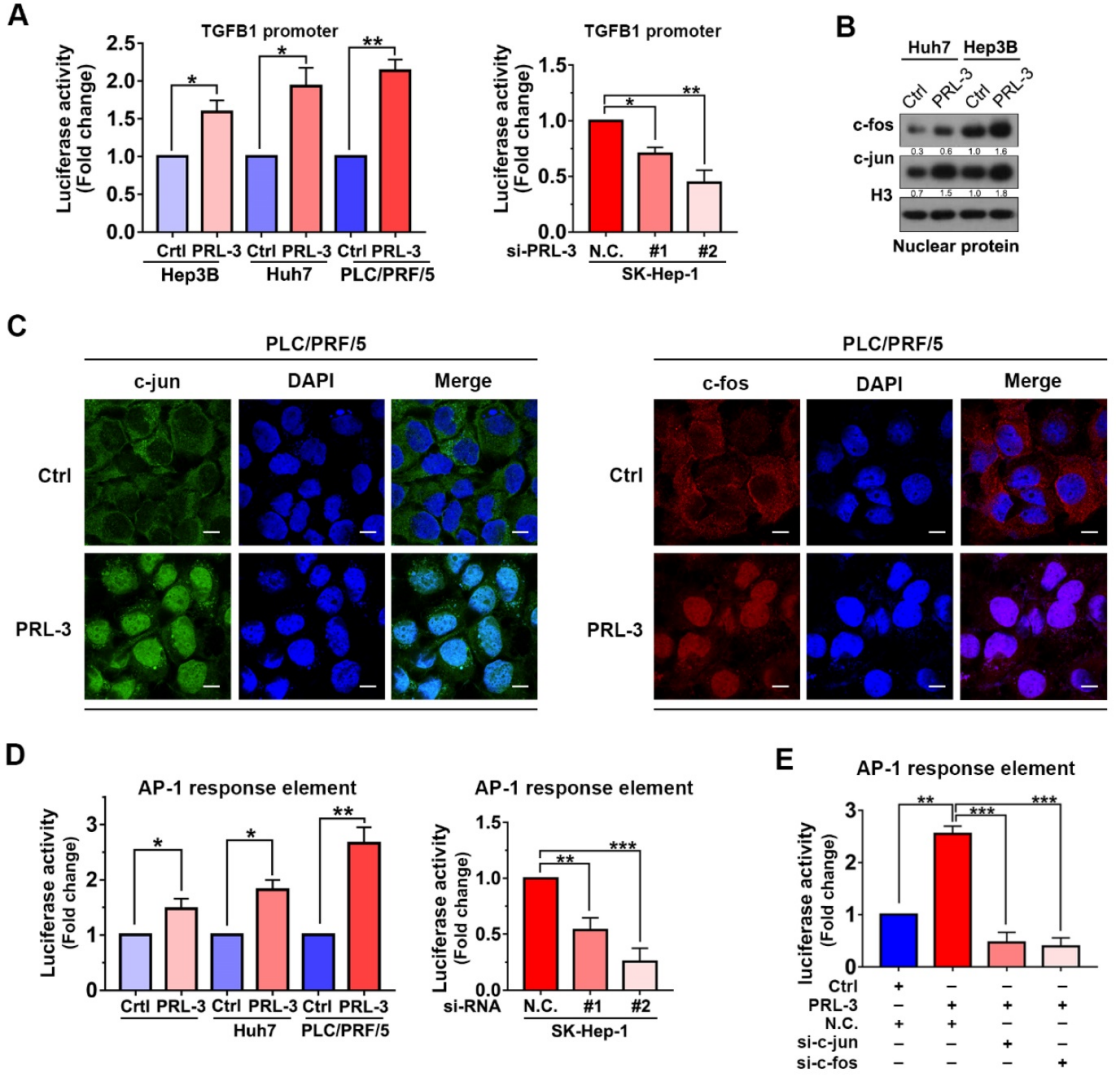
** PRL-3 enhances TGFB1 transcription and AP-1 complex activation. (A)** Dual-luciferase reporter assay of the indicated cells cotransfected with the luciferase reporter vector for the TGFB1 gene. **(B)** The nuclear expression of c-jun and c-fos in PRL-3-overexpressing and control cells, as determined by western blot.** (C)** Immunofluorescence staining showing translocalization of c-jun and c-fos under PRL-3 overexpression conditions. Scale bar: 100 µm. **(D)** Dual-luciferase reporter assay of the indicated cells cotransfected with the AP-1 reporter vector. **(E)** Dual-luciferase reporter assay of PLC/PRF/5 cells cotransfected with the AP-1 reporter vector and c-jun- or c-fos siRNA. The results are presented as the means ± standard error of the mean of three independent experiments. NS, not significant, *, *P* < 0.05, **, *P* < 0.01, ***, *P* < 0.001.

As our above results showed that both mRNA and protein expression of TGFB1 were regulated by PRL-3, we next investigated whether PRL-3 regulates TGFB1 expression at the transcriptional level. We observed that PRL-3 overexpression significantly enhanced TGFB1 gene promoter activity, while PRL-3 knockdown had the opposite effect (Figure 7A). The transactivation of the TGFB1 gene has been widely reported to be mediated by AP-1 24, and c-jun and c-fos are the two key members of the AP-1 complex 25. Our western blot results showed that PRL-3 overexpression in Huh7 and Hep3B cells increased the expression of c-jun and c-fos in the nucleus (Figure 7B). Immunofluorescence staining results further confirmed the translocation of c-jun and c-fos from cytoplasm to nucleus in PRL-3 overexpressing cells (Figure 7C). Moreover, the AP-1 response element was activated by PRL-3 overexpression, but inhibited by PRL-3 knockdown (Figure 7D), and of c-jun and c-fos abrogated the ability of PRL-3 to activate AP-1 response element (Figure 7E). These results suggested that AP-1 complex may be a functional executor of PRL-3 in HCC.

**Figure 8:**
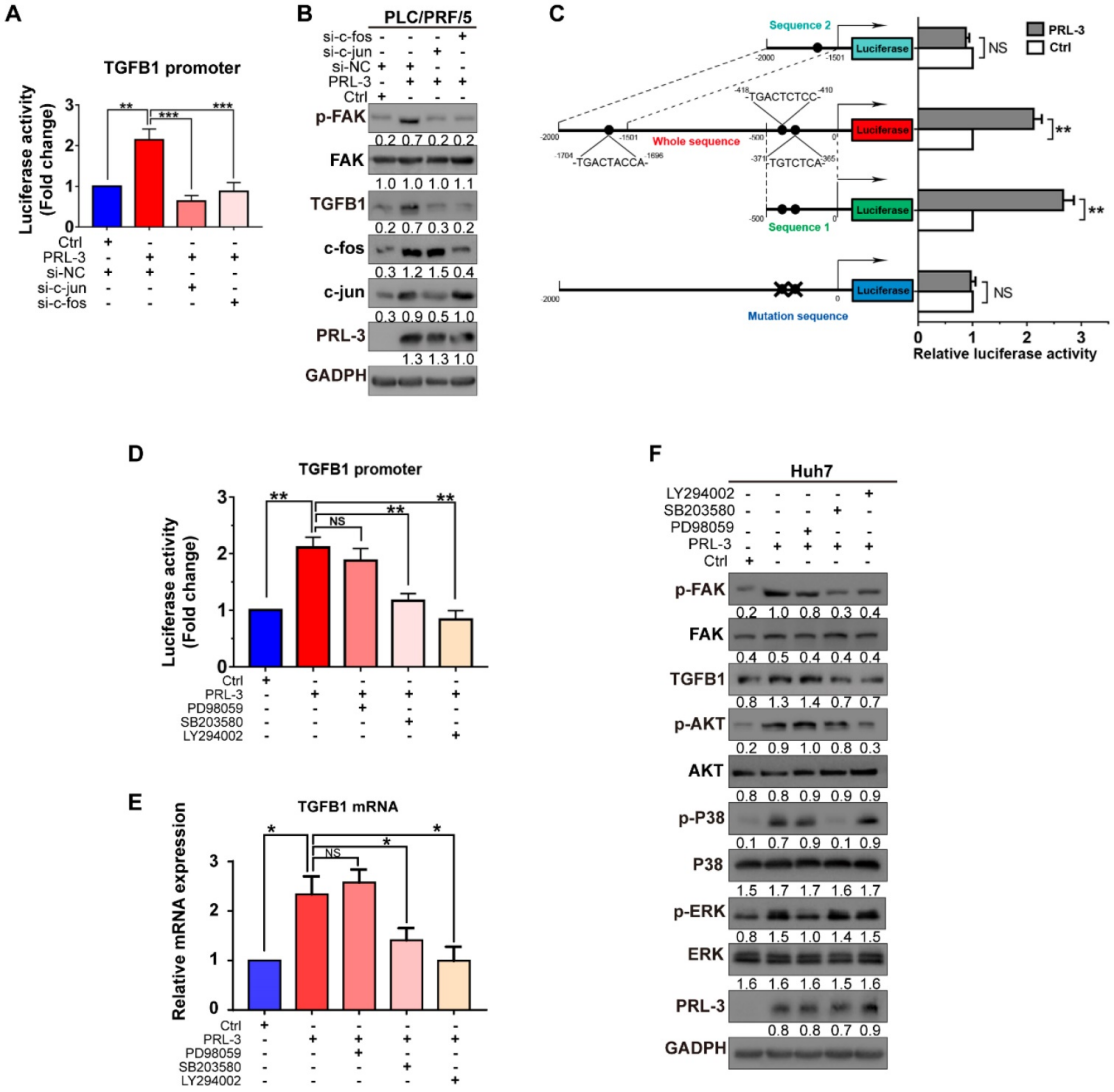
** p38 and PI3K/AKT mediate PRL-3-induced AP-1/TGFB1 transcription. (A)** Dual-luciferase reporter assay detection of the changes in TGFB1 promoter transcriptional activity after PRL-3-overexpressing PLC/PRF/5 cells were transfected with c-jun- or c-fos siRNA. (**B**) Western blot detection of the changes in FAK activation and the protein expression of TGFB1 after PRL-3-overexpressing PLC/PRF/5 cells were transfected with c-jun- or c-fos siRNA. **(C)** Dual-luciferase reporter assay of PLC/PRF/5 cells cotransfected with the indicated luciferase reporter. Schematic representation (left panel) showed the sizes of the flanking insert in the TGFB1 chimeric gene promoter and the mutation sites within the TGFB1 promoter region. (**D**) Dual-luciferase reporter assay of PLC/PRF/5 cells cotreated with TGFB1 promoter vector and an ERK inhibitor (PD98059: 10 µM, 24 h), p38 inhibitor (SB203580: 10 µM, 24 h), or PI3K/AKT pathway inhibitor (LY294002: 10 µM, 24 h). (**E-F**) Huh7 cells overexpressing PRL-3 were also treated with ERK, p38 and PI3K/AKT pathway inhibitors, respectively (10 µM, 24 h). (**E**) qPCR was performed to examine the mRNA expression of TGFB1, and (**F**) western blot analysis was performed to detect the changes in FAK activation and TGFB1 protein expression of. The results are presented as the means ± standard error of the mean of three independent experiments. NS, not significant, *, *P* < 0.05, **, *P* < 0.01.

We then examined whether PRL-3 promotes TGFB1 transcription through the AP-1 complex. The results showed that c-jun and c-fos knockdown reduced not only PRL-3-enhanced TGFB1 promoter activity but also PRL-3-dependent TGFB1 expression and FAK activation (Figure 8A and 8B). The presence of two AP-1 binding sites within the TGFB1 promoter region (from -323 to -453 bp) has been reported 26, and the PROMO online program also predicted another AP-1 binding site in the TGFB1 promoter region (from -1696 to -1704) bp 27. We generated reporter constructs for these two regions [sequence 1 (0 to -500 bp) and sequence 2 (-1500 to -2000 bp)] and observed that PRL-3 overexpression enhanced expression from sequence 1 but not sequence 2 (Figure 8C). Moreover, the expression was abolished when the AP-1 binding sites within sequence 1 were mutated (Figure 8C). Taken together, these results indicate that PRL-3 enhances TGFB1 promoter activity through the AP-1 complex.

### PRL-3 regulates a positive feedback loop comprising PI3K/AKT or p38, TGF-β and FAK in HCC

Because the transcriptional activity of AP-1 could be controlled by either MAPK family (including ERK, JNK and p38) or PI3K/AKT pathways 28, we next assessed whether one or more of these signalling molecules were involved in PRL-3-induced AP-1 activation and subsequent TGFB1 expression. The results showed that PRL-3 overexpression increased the phosphorylation of p38, ERK and AKT, but not JNK (Figure S6A), whereas the overexpression of a catalytically inactive PRL-3 mutant (PRL-3-C104S) abrogated these effects (Figure S6B). We then used specific inhibitors to inhibit the p38, ERK and AKT signalling pathways, and the results showed that all three inhibitors attenuated PRL-3-induced AP-1 response element activation (Figure S6C and S6D). However, only inhibition of the p38 (SB203580) or AKT (LY294002) pathways decreased the TGFB1 promoter activity induced by PRL-3 (Figure 8D). RT-qPCR and western blot results further confirmed that inhibition of the p38 or AKT pathways decreased PRL-3-dependent TGFB1 expression and FAK activation (Figure 8E, F). These results suggest that PRL-3 promotes AP-1 activation and the subsequent TGFB1 upregulation through the PI3K/AKT and p38 signalling pathways.

Since p38 and PI3K/AKT are also well-known signal transduction elements that function downstream of FAK 29, 30, we next evaluated whether there is positive feedback between the PI3K/AKT or p38 pathways and activated FAK. We observed that inhibition of FAK phosphorylation by PND-1186 reduced activation of the PI3K/AKT and p38 pathways and decreased downstream TGFB1 expression but did not affect PRL-3 expression (Figure S6E). However, when PRL-3 was overexpressed, inhibition of FAK activation only partially attenuated PI3K/AKT and p38 pathway activation (Figure S6F). These results, together with our above data, suggested that there exists a positive feedback loop comprising PI3K/AKT or p38, TGF-β and FAK in HCC and that PRL-3 is an upstream regulator which can trigger this positive feedback loop through activating PI3K/AKT and p38.

## Discussion

HCC is one of the leading causes of cancer-related deaths worldwide 31. Most HCC patients are diagnosed at an advanced stage and have no chance of surgical resection 32. PTK inhibitors, such as sorafenib and regorafenib, are recommended for advanced stage HCC patients 33. However, the survival benefits these drugs offer patients remain unsatisfactory. In addition to PTKs, PTPs are also crucial for regulating protein phosphorylation. During normal cellular processes, kinases and phosphatases work concertedly to ensure normal signal transmission 34. However, dysregulation of either PTKs or PTPs can result in the aberrant phosphorylation of multiple downstream proteins, which is a well-recognized cause of cancer 2, 35. Therefore, additional focus should also be paid to PTPs for their therapeutic potential in HCC. In the present study, we showed that a well-known PTP, PRL-3, which has been well studied in many malignant tumours, is involved in promoting HCC 36. In fact, some studies have shown that PRL-3 is upregulated in HCC tissues and contributes to HCC progression through the PI3K/AKT pathway 5, 37. In this study, we observed that PRL-3 was upregulated in HCC due to its gene amplification. PRL-3 overexpression in HCC tissues was positively correlated with a larger tumour size, increased vascular invasion and advanced TNM stage and was associated with poorer survival. Mechanistically, PRL-3 was shown to promote HCC cell proliferation, migration and adhesion to ECM substrates by activating the TGFB1/Src/FAK pathway. Furthermore, we observed that PRL-3 triggered a positive feedback loop comprising PI3K/AKT or p38, TGF-β and FAK in HCC. We thus provide a more complete data indicating that PRL-3 acts as a tumour promoter in HCC.

In the present study, we initially observed that PRL-3 expression was positively correlated with FAK expression at both the mRNA and protein levels. Interestingly, our subsequent findings suggested that this co-expression did not result from the mutual regulation of PRL-3 and FAK at either the mRNA or protein expression level but rather was due to their co-amplification on chromosome 8q24.3. Gene co-amplification results in the overexpression of a number of neighbouring genes, where some of them can cooperate to promote tumour initiation and progression 8. However, the cooperation mechanism varies under different circumstances. Saladi et al. reported that ACTL6A and p63 are co-amplified in head and neck squamous cell carcinoma and form a protein complex with each other to promote tumour progression 10. A similar mechanism was also shown to occur for PIKE-A and Cdk4 in glioblastoma 38. In contrast, S100A7, S100A8 and S100A9, all of which are co-amplified on chromosome 1q21.3 in breast cancer, have similar functions and work together to induce target protein phosphorylation 9. In addition to coding genes, long non-coding RNAs (lncRNAs) are also well-known to play a regulatory role in the transcription of its neighbouring genes, which is called in* cis* regulation 39, 40. Our data showed that PRL-3 regulates FAK phosphorylation through the TGFB1/Src signalling pathway rather than by directly interacting with FAK (data not shown) or regulating FAK expression. Thus, different mechanisms are involved in the effects of “co-amplified genes” associated with tumour-promoting processes. Thus, our results may advance the understanding of how co-amplified genes function in malignant tumours.

The function of TGF-β signalling is paradoxical in HCC. In the early stage of HCC, TGF-β inhibits tumourigenesis through the physiological response by inducing cell cycle arrest and apoptosis. In contrast, in the advanced stage of HCC, TGF-β can increase tumour invasion and recurrence 41. It is believed that genetic context is one reason why TGF-β has different effects on tumourigenesis. Martin Oft et al. reported that in the process of tumour progression, the coactivation of smad2 and H-ras can weaken the antitumour effect of TGF-β, leading to tumour invasion and metastasis 42. Maddalena Adorno et al. showed that TGF-β-dependent tumour progression is promoted in the presence of mutant p53, which can intercept p63 (an antagonist of TGF-β) to drive the TGF-β-mediated promotion of tumour invasiveness and metastasis 43. Francisco Valdés et al. showed that the PI3K/AKT pathway is activated in cells resistant to TGF-β-induced apoptosis. Inhibiting the PI3K/AKT pathway sensitizes foetal hepatocytes (FH) to the apoptosis induced by TGF-β and causes spontaneous death in the resistant cells 44. Overall, the results of these studies indicate that the role of TGF-β in tumours may depend on the concurrent gene alterations or signalling pathway activation status in tumour cells. We supposed that in the cell model of our study, the PRL-3-mediated induction of PI3K/AKT pathway activation together with the activation of FAK induced by PRL-3-TGF-β may generate the tumour-promoting genetic context for TGF-β. These data further suggesting the important role of PRL-3 in HCC.

## Conclusions

The results of the present study indicate that PRL-3 is commonly amplified and overexpressed in HCC and that FAK is co-expressed with PRL-3 due to co-amplification on chromosome 8q24.3 in HCC. Furthermore, we identified TGFB1 as a novel downstream molecule of PRL-3 in HCC. TGFB1 enhances FAK phosphorylation, promoting HCC cells proliferation, migration and adhesion to ECM substrates. We also elucidated a PRL-3-triggered AKT/p38/TGFB1/FAK positive feedback loop in HCC. Thus, our data indicate that PRL-3, as a PTP, may be a potential therapeutic target for HCC. In addition, our results also provide an example of how co-amplified genes work together malignant tumours.

## Supplementary Material

Supplementary figures and tables.

## Abbreviations

PRL-3: phosphatase of regenerating liver-3
TGFB1: transforming growth factor beta 1
FAK: focal adhesion kinase
GAPDH: glyceraldehyde-3-phosphate dehydrogenase
MTT: 3-(4,5-dimethyl-2-thiazolyl)-2,5-diphenyl-2-H-tetrazolium bromide
DMSO: dimethyl sulfoxide
CCK-8: Cell Counting Kit-8
HE: Haematoxylin-eosin
PBS: phosphate buffer saline
BSA: bovine serum albumin
ELISA: enzyme linked immunosorbent assay
DAPI: 4',6-diamidino-2-phenylindole
