# Supplementary material for: PRL-3 facilitates Hepatocellular Carcinoma progression by co-amplifying with and activating FAK
Source: Theranostics. 2020 Aug 18;10(22):10345–59. doi: 10.7150/thno.42069 (PMC7481414; doi:10.7150/thno.42069)
Supplement: Supplementary file 1 — Supplementary figures and tables. [file thnov10p10345s1.pdf]

Supplementary Figure

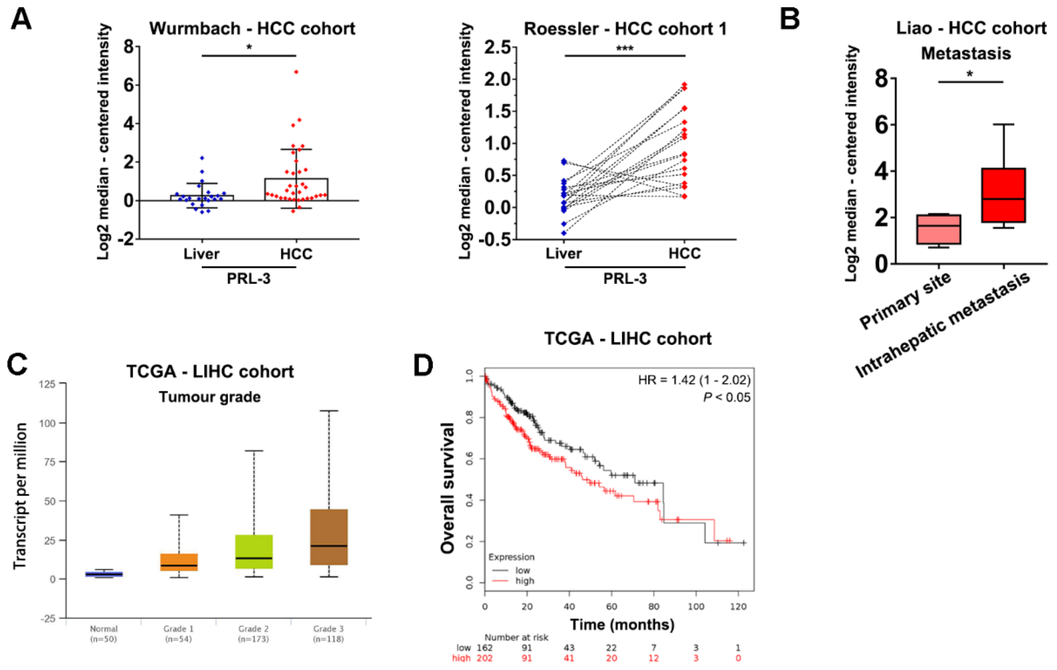

Figure S1. Bioinformatic analysis of the expression level and prognosis value of PRL-3 in HCC. **(A)** Bioinformatic analysis of mRNA expression level of PRL-3 in Wurmbach-HCC cohort and Roessler-HCC cohort 1. Data were obtained from ONCOMINE database (www.Oncomine.Org). **(B)** Bioinformatic analysis and comparison of mRNA expression level of PRL-3 in the HCC patients grouped by the tumour sites in Liao-HCC cohort. Data were obtained from ONCOMINE database (www.Oncomine.Org). **(C)** Bioinformatic analysis and comparison of mRNA expression level of PRL-3 in HCC patients grouped by tumour grade in TCGA-LIHC cohort. Data were obtained from UALCAN cancer database (http://ualcan.path.uab.edu/). **(D)** Kaplan-Meier overall survival curve of two HCC groups in TCGA-LIHC cohort: High PRL-3 expression (red) and low PRL-3 expression (black). Data were obtained from the Kaplan–Meier (KM) plotter integrative data analysis tool (http://www.kmplot.com) and the two groups were grouped by the best cut-off value obtaining on the website (http://www.kmplot.com). * $P < 0.05$; ** $P < 0.01$; *** $P < 0.001$.

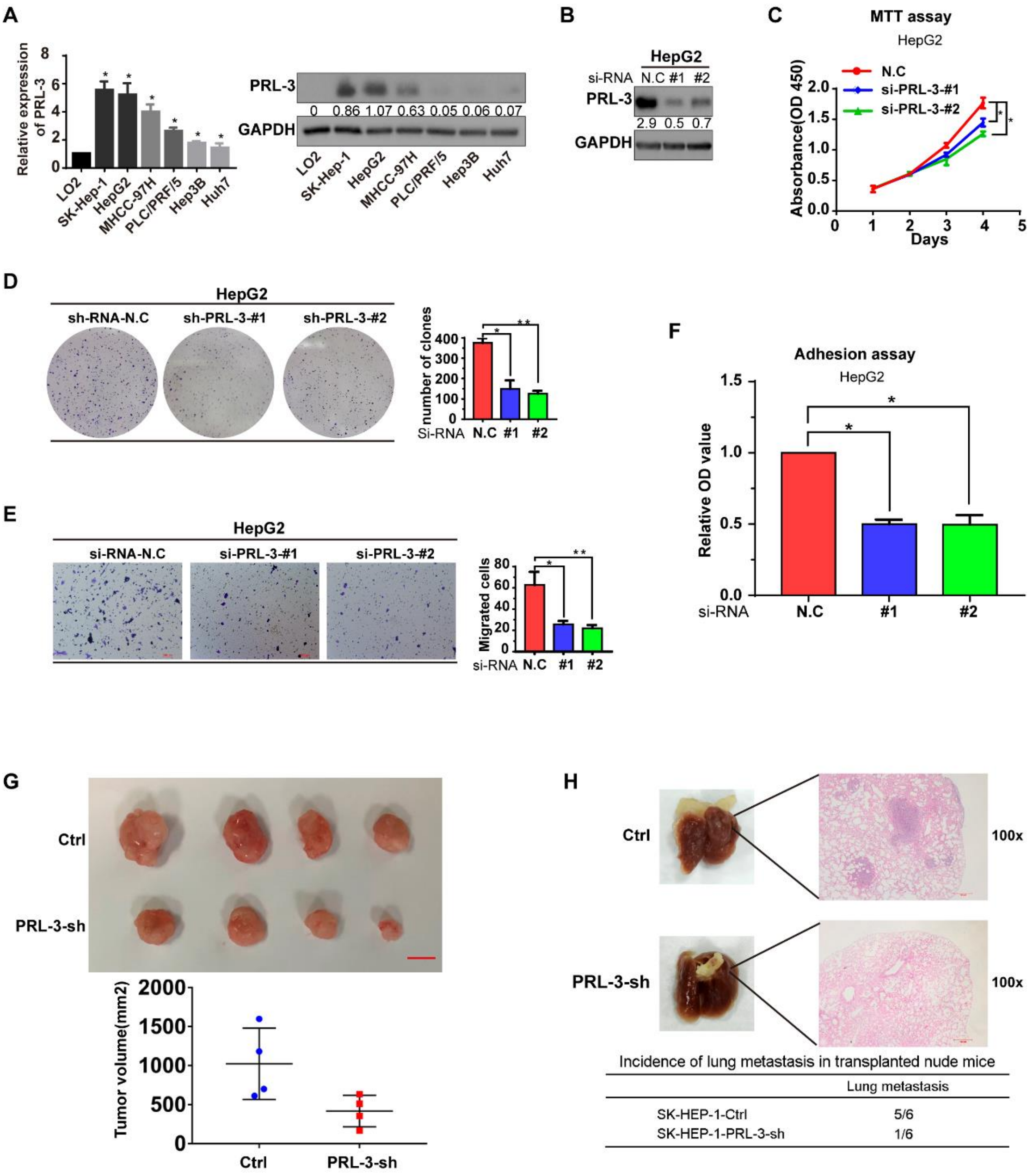

Figure S2. Downregulation of PLR-3 inhibits HCC cells proliferation and metastasis in vitro and vivo. (A) The mRNA and protein expression pattern of endogenous PRL-3 in HCC cell lines and normal control cells were detected. **(B)** PRL-3 knockdown in Hep3B was confirmed by western blot. **(C-F)** MTT **(C)**, clone formation **(D)**, Transwell **(E)** and cell-matrix adhesion assay**(F)** of Hep3B cells with PRL-3 knockdown. **(A-F)** The results are expressed as the means ± standard error of the mean of three independent experiments. * $P < 0.05$, ** $P < 0.01$. **(G)**

Tumours derived from nude mice subcutaneously implanted with SK-Hep-1-Ctrl or SK-Hep-1-PRL-3-sh cells. Scale bar: 1 cm. Tumour volume are shown as means ± SEM. **(H)** Lungs derived from nude mice with tail intravenous injection of SK-Hep-1-Ctrl or SK-Hep-1-PRL-3-sh cells and the corresponding H&E staining images. Scale bar: 100 um. * $P < 0.05$.

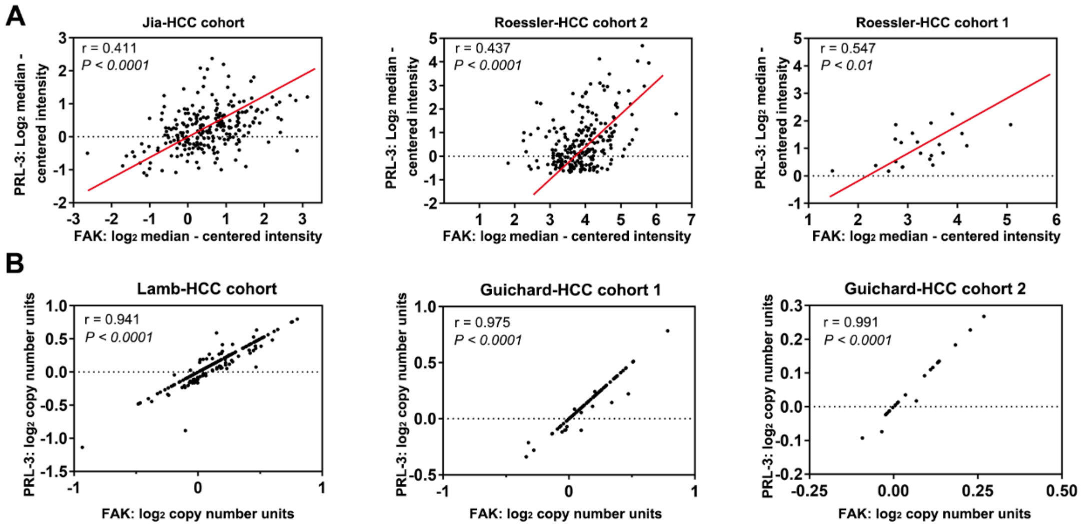

Figure S3. Positive correlation between PRL-3 and FAK in mRNA expression and copy number variations. (A) Positive correlation between PRL-3 and FAK mRNA expression in Jia-HCC cohort, Roessler-HCC cohort 2 and Roessler-HCC cohort 1. Data were obtained from ONCOMINE database (www.oncomine.org). **(B)** Positive correlation in copy number variations between PRL-3 and FAK gene in Lamb-HCC cohort, Guichard-HCC cohort 1 and Guichard-HCC cohort 2. Data were obtained from ONCOMINE database (http://www.oncomine.org).

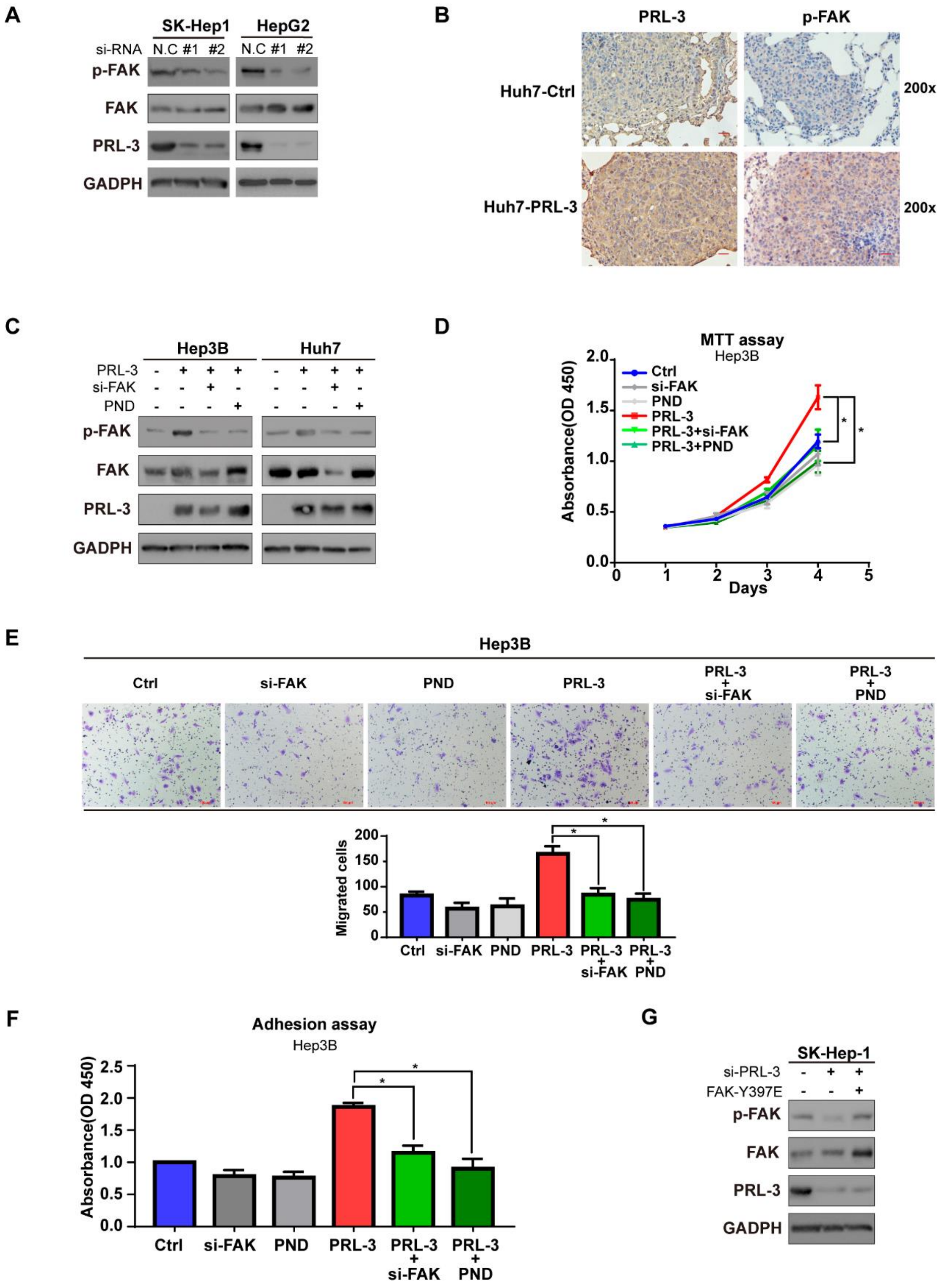

Figure S4. PRL-3 functions through phosphorylating FAK. (A) Western blot showed knockdown of PRL-3 decrease FAK phosphorylation in SK-Hep-1 and HepG2 cells. **(B)**The expression of PRL-3 and phosphorylated FAK in metastasis tumours of mice was detected by

immunohistochemistry. Scale bar: 10 um. **(C)** The knockdown efficiency of FAK siRNA and the inhibition efficiency of FAK inhibitor PND-1186 were examined by western blot. PND-1186(0.1 μM) for 24h. **(D, E, F)** The effect of FAK knockdown or inhibition on Hep3B cells with PRL-3 overexpression were evaluated by MTT**(D)**, Transwell**(E)** and cell adhesion assay**(F)**. **(G)** Western blot was performed to confirmed FAK was continuously activated in SK-Hep-1 cells overexpressed FAK-Y397E mutant. Data represent mean and SD of three independent experiments. * $P < 0.05$.

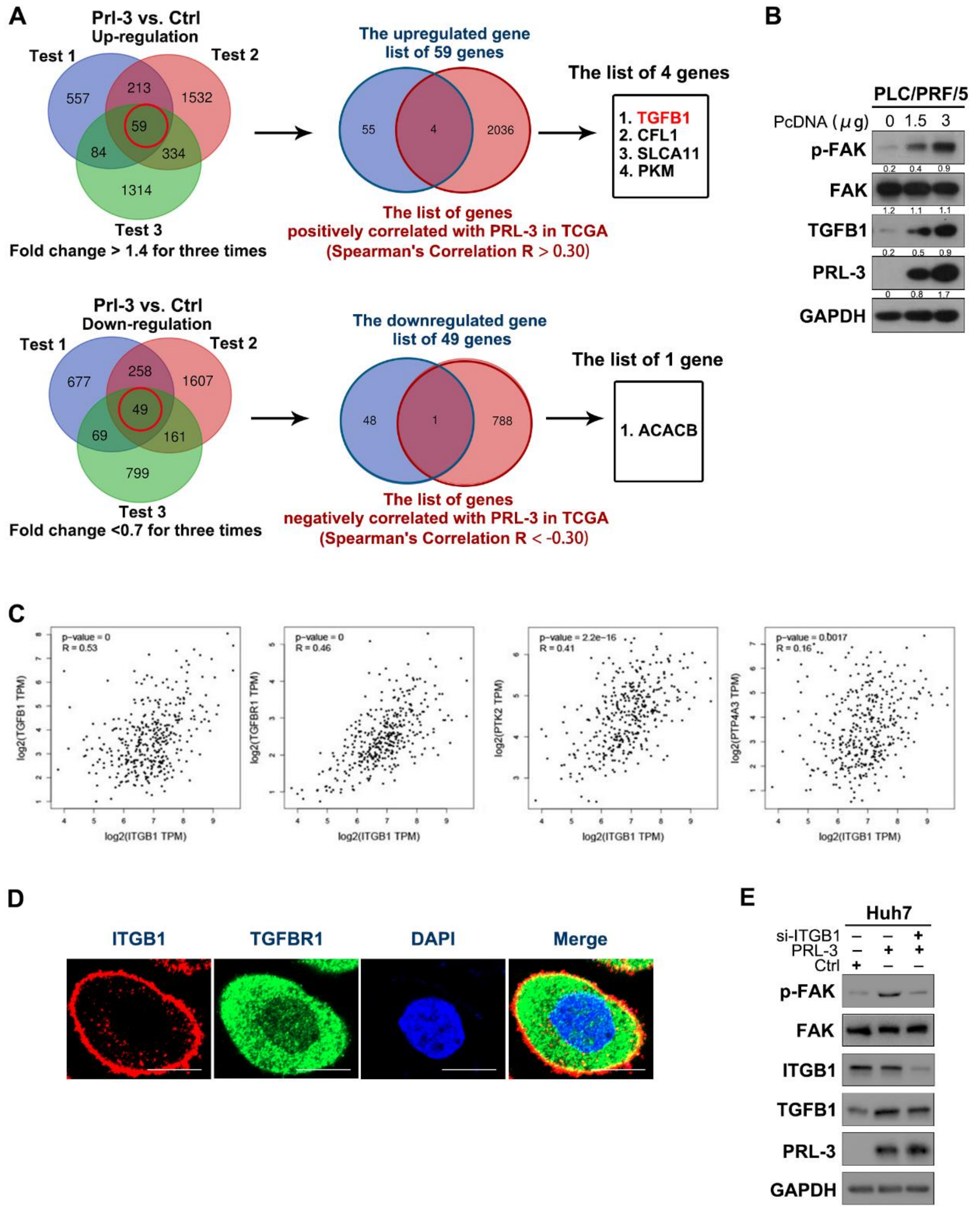

Figure S5. A crosstalk of TGF-β/TGFBR1 with integrin β1 is involved in PRL-3 activating FAK. (A) Venn diagram (Left) showed genes that had consistent expression pattern within three gene microarray assays (Fold change > 1.4 or < 0.7 for three times, $P < 0.05$). Venn diagram (Middle) and table (Right) showed genes that had consistent expression pattern in our mRNA microarray data and genes list correlated with PRL-3 expression in TCGA-LIHC dataset (R > 0.30

or R < -0.30, $P < 0.05$, Spearman's correlation analysis). Data were obtained from cBioPortal database (http://www.cbioportal.org/). **(B)** The regulation of TGFB1 by PRL-3 was confirmed in PLC/PRF/5 cells transiently transfected with PRL-3, as determined by western blot with indicated antibodies. **(C)** TCGA-LIHC dataset showed ITGB1(integrin β1) has a significant correlation with FAK, PRL-3, TGFB1 and TGFBR1 in HCC**.** Data was derived from GEPIA database (http://gepia.cancer-pku.cn/index.html). **(D)** Immunofluorescent staining displayed integrin β1 and TGFBR1 co-localized in the cell membrane. **(E)** Western blot revealed knockdown of integrin β1 inhibited the PRL-3-induced activation of FAK.

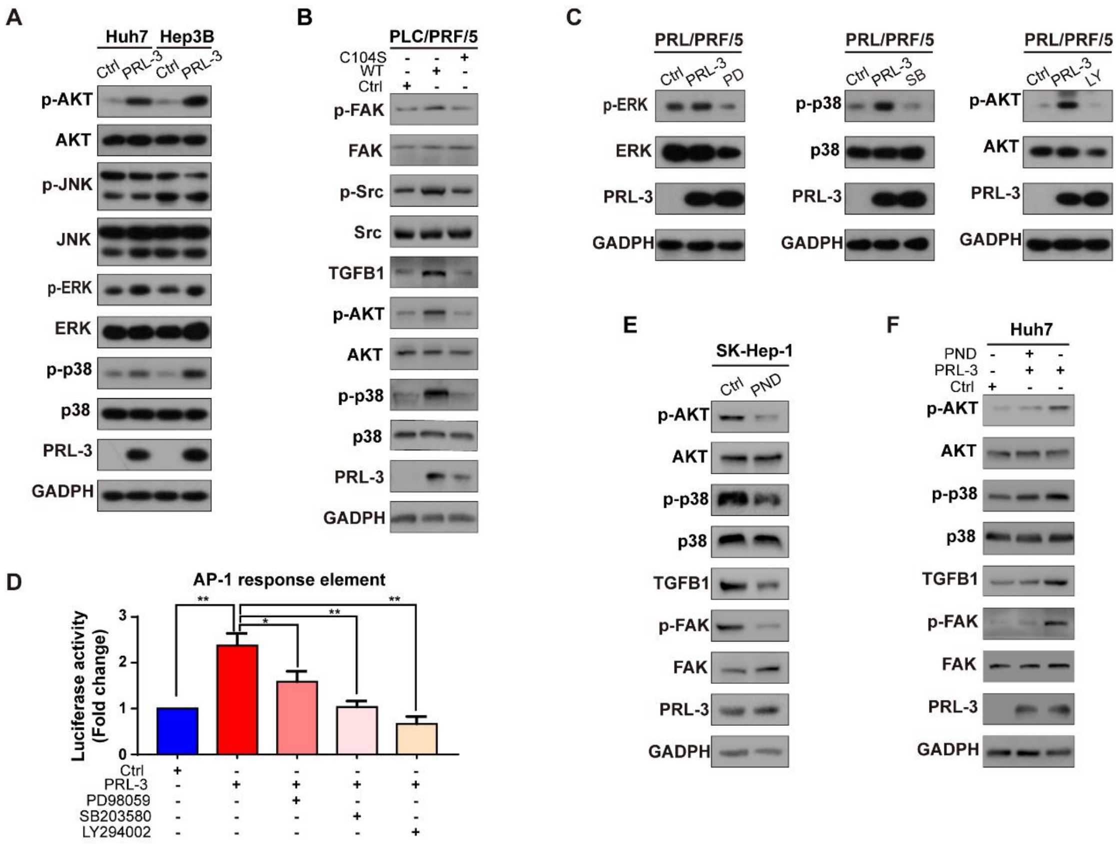

Figure S6. PRL-3 triggers a positive feedback loop through activating PI3K/AKT and p38.

(A) Activities of MAPK and PI3K signaling in the PRL-3 overexpression group and its control group, as determined by western blots with indicated antibodies. **(B)** PLC/PRF/5 cells were overexpressed catalytically inactive PRL-3 mutant (C104S) or wild type PRL-3(WT), and activation of the pathway regulated by PRL-3 was detected by western blot. (**C**) The inhibitory effects of PD98059, SB203580 and LY294002 under the condition of PRL-3 overexpression. **(D)** Dual-luciferase reporter assay of PLC/PRF/5 cells cotreated with AP-1 reporter vector and PD98059, SB203580 or LY294002. **(E)** The effect of FAK phosphorylation inhibitor PND-1186 on PI3K/AKT, p38 pathway and TGFB1. **(F)** The effect of PND-1186 on PRL3 overexpressed Huh7 cells. (**C-F**) The concentrations and duration of inhibitors PD98059, SB203580, LY294002 was 10 uM for 24h, and PND-1186 was 0.1 μM for 24h. The results are showed as the means ± standard error of the mean of three independent experiments. * $P < 0.05$, ** $P < 0.01$, *** $P <$

0.001.

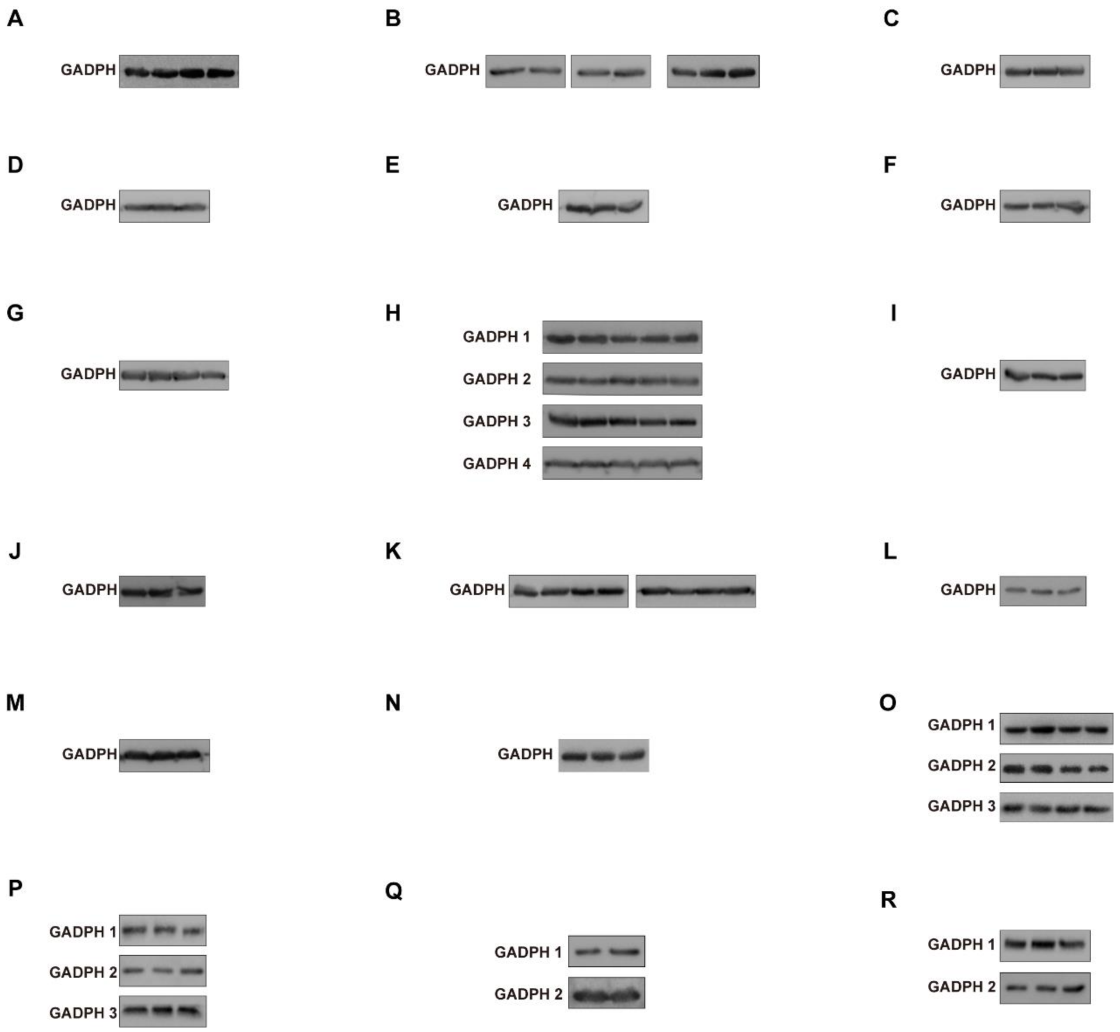

Figure S7. Corresponding GAPDH control bands for western blot. (A) GADPH band corresponding to Paxillin and FAK in Figure 5A. **(B)** Three GADPH bands corresponding to p-FAK and p-Src in Figure 6C. **(C)** GADPH band corresponding to p-FAK and p-Src in Figure 6F **(D)** GADPH band corresponding to p-FAK and p-Src in Figure 6G. **(E)** GADPH band corresponding to p-FAK and p-Src in Figure 6H. **(F)** GADPH band corresponding to p-FAK and p-Src in Figure 6I. **(G)** GADPH band corresponding to p-FAK and c-jun in Figure 8B. **(H)** GADPH 1 was a control band corresponding to FAK, AKT and p38, GADPH 2 was a control band corresponding to TGFB1, GADPH 3 was a control band corresponding to p-P38 , GADPH 4 was a control band corresponding to ERK in Figure 8F. **(I)** GADPH band corresponding to p-FAK in Figure S4A(left) f. (J) GADPH band corresponding to p-FAK in Figure S4A(right). **(K)** GADPH

band corresponding to p-FAK in Figure S4C. **(L)** GADPH band corresponding to p-FAK in Figure S4G. **(M)** GADPH band corresponding to p-FAK in Figure S5B. **(N)** GADPH band corresponding to p-FAK in Figure S5E. **(O)** GADPH 1 was a control band corresponding to JNK and ERK, GADPH 2 was a control band corresponding to p-AKT and p-p38, GADPH 3 was a control band corresponding to AKT and p38 in Figure S6A. **(P)** GADPH 1 was a control band corresponding to FAK and AKT, GADPH 2 was a control band corresponding to p-Src and p38, GADPH 3 was a control band corresponding to Src, TGFB1 and p-p38 in Figure S6B. **(Q)** GADPH 1 was a control band corresponding to p-AKT, p38 and p-FAK, GADPH 2 was a control band corresponding to p-p38 in Figure S6E. **(R)** GADPH 1 was a control band corresponding to p-AKT, TGFB1 and p-FAK, GADPH 2 was a control band corresponding to p38 in Figure S6F.

Supplementary Table

Table 1 Association of PRL-3 expression levels with clinicopathologic characteristics in HCC

Clinicopathologic Features	Total	PRL-3 expression level		*P* value
		Low Counts (n%)	High Counts (n%)	
Age, years				
< 55	45	19 (46.3)	26 (47.3)	0.928
≥ 55	51	22 (53.7)	29 (52.7)	
Gender				
Female	29	12 (29.3)	17 (30.9)	0.863
Male	67	29 (70.7)	38 (69.1)	
AFP level				
≤ 20	26	10 (24.4)	16 (29.1)	0.608
> 20	70	31 (75.6)	39 (70.9)	
HBV infection				
Negative	15	9 (22.0)	6 (10.9)	0.141
Positive	81	32 (78.0)	49 (89.1)	
Tumour size (cm)				
< 5	34	22 (53.7)	12 (21.8)	0.001 (**)
≥ 5	62	19 (46.3)	43 (78.2)	
Tumour number				
Single	74	29 (70.7)	45 (81.8)	0.201

Multiple	22	12 (29.3)	10 (18.2)	
Vascular invasion				
Absent	44	25 (61.0)	19 (34.5)	0.010 (*)
Present	52	16 (39.0)	36 (65.5)	
Tumour capsule				
Absent	49	23 (56.1)	26 (47.3)	0.392
Present	47	18 (43.9)	29 (52.7)	
TNM stage				
I	31	20 (48.8)	11 (20.0)	0.003 (**)
II + III	65	21 (51.2)	44 (80.0)	

AFP alpha fetoprotein,, HBV hepatitis B virus, TNM tumour-node-metastasis

p values were calculated by comparing the expression of PRL-3 with different clinical variables respectively using a chi-square test. $p < 0.05$ was considered statistically significant. *$P < 0.05$; **$P < 0.01$;

Supplementary Materials and Methods

The si-RNA sequence list

PRL-3	Si-RNA-#1	Forward, 5′-GCUACAAACACAUGCGCUUTT-3′<br>Reverse, 5′-AAGCGCAUGUUUGUAGCTT -3′
	Si-RNA-#2	Forward, 5′-GGAGAAAUACCGGCCCAAATT-3′<br>Reverse, 5′-UUUGGGCCGGUAUUUCUCCTT-3′
FAK	Si-RNA-#3	Forward, 5′-CAGGUGAGCGAUUAUATT-3′<br>Reverse, 5′-UAUAAUCGCUCUUCACCUGTT-3′
	Si-RNA-#4	Forward, 5′-GGGCAUCAUUCAGAAGAUATT-3′,<br>Reverse, 5′-UAUCUUCUGAAUGAUGCCCTT-3′
c-Jun	Si-RNA	Forward, 5′-GCAAACCUCAGCAACUUCATT-3′,<br>Reverse, 5′-UGAAGUUGCUGAGGUUUGCTT-3′.
c-Fos	Si-RNA	Forward, 5′-GGGAUAGCCUCUUACUATT-3′,<br>Reverse, 5′-UAGUAAGAGAGGCUAUCCCTT-3′.
Src	Si-RNA	Forward, 5′-CUGUAUCCGACUUCGACAATT-3′<br>Reverse, 5′-UUGUCGAAGUCGGAUACAGTT-3′

The primer sequence list

PRL-3	Forward	5′-CACATGCGCTTCCTCATCAC-3′
	Reverse	5′-CCAGCGGCGTTTTGTCATAG-3′
GAPDH	Forward	5′-ATTCCACCCATGGCAAATTCC-3′
	Reverse	5′-GGGCAGAGATGATGACCCTT-3′.

TGFB1	Forward	5′-GGCCTTTCCTGCTTCTCATG-3′
	Reverse	5′-GCAGAAGTTGGCATGGTAGC-3′

The list of antibodies for western blot

Anti-Homo sapiens primary antibodies			
Antibodies	Dilution	Identifiter	Source
Mouse anti-human GAPDH	1:10000	60004-1-Ig	Proteintech, USA
Rabbit anti-human PRL-3	1: 1000	ab50276	Abcam, Cambridge, UK
Rabbit anti-human FAK	1: 1000	#71433	Cell Signaling Technology, MA, USA
Rabbit anti-human phospho-FAK	1: 1000	ab38512	Abcam, Cambridge, UK
Rabbit anti-human Paxillin	1: 1000	YT3605	ImmunoWay, USA
Rabbit anti-human phospho-Paxillin	1: 1000	YP0219	ImmunoWay, USA
Rabbit anti-human TGFB1	1:2000	YT4632	ImmunoWay, USA
Rabbit anti-human Src	1:1000	#2108	Cell Signaling Technology, MA, USA
Rabbit anti-human phosphor-Src	1:1000	#6943	Cell Signaling Technology, MA, USA
Rabbit anti-human c-jun	1: 1000	#9165	Cell Signaling Technology, MA, USA
Rabbit anti-human c-fos	1: 1000	ab214672	Abcam, Cambridge, UK
Mouse anti-human AKT	1:1000	#2966	Cell Signaling Technology, MA, USA
Rabbit anti-human phosphor-AKT	1: 1000	#4060	Cell Signaling Technology, MA, USA
Rabbit anti-human ERK	1:1000	#4695	Cell Signaling Technology, MA, USA
Rabbit anti-human p38	1:1000	#8690	Cell Signaling Technology, MA, USA
Rabbit anti-human phosphor-p38	1:1000	#4511	Cell Signaling Technology, MA, USA

Rabbit anti-human JNK	1: 1000	#9252	Cell Signaling Technology, MA, USA
Rabbit anti-human phospho-JNK	1:1000	#4671	Cell Signaling Technology, MA, USA
Mouse anti-human ITGB1	1:1000	Ab24693	Abcam, Cambridge, UK
Secondary antibodies for western blot			
Goat anti-mouse	1:10,000	sc-2005	Santa Cruz Biotechnology, TX USA
Goat anti-rabbit	1:10,000	sc-2004	Santa Cruz Biotechnology, TX USA

Inhibitors, growth factors, cell lines and transfection

FAK inhibitor PND-1186 and TGF-β receptor SB-431542 were purchased from Medchem Express, co (Monmouth Junction, NJ, USA). ERK inhibitor PD98059, p38 inhibitor SB203580, and PI3K/AKT pathway inhibitor LY294002 were purchased from APExBIO, Technology (Houston, USA).

Human Recombinant TGF-β1 was purchased from PeproTech (CT, USA).

All cell lines (293T, PLC/PRF/5, Sk-hep-1, Huh7, Hep3B) were purchased from Cell Bank in Chinese Academy of Sciences (Shanghai, China) and were cultured in DMEM supplemented with 10% fetal bovine serum and 1% antibiotics at 37 °C in a humidified incubator with 5% CO2. (all reagents from Thermo Fisher Scientific, Waltham, MA, USA).

The PRL-3 open reading frame (GenBank: NM_007079.3) was cloned into the lentiviral vector pCDH (GENEray, Shanghai, China). Lentiviral particles delivering pCDH-control or pCDH-PRL-3 was generated from 293T cells and was used to infect Huh7 cells and Hep3B cells. Selective culture medium containing 2 μg/mL puromycin was used to select cells with stable expression of PRL-3 for 2 weeks. Stable expression of PRL-3 in HCC cells was detected by RT-qPCR and

western blot analysis. All small interfering RNAs (siRNAs) were constructed by GenePharma company (Shanghai, China). The cells were transfected siRNAs by using Lipofectamine 3000 (Thermo Fisher Scientific, MA, USA) according to the manufacturer's protocols. Lentivirus constructed from PRL-3 shRNAs (Genechem company, Shanghai, China) were transfected into the SK-Hep-1 and HepG2 cells and then selected by 2 μg/mL puromycin for 2 weeks to establish stable cells. The plasmids of PRL-3-C104S mutant, FAK-Y397E mutant and Src-Y527F mutant was constructed and sequenced by IGE biotechnology company (Guangzhou, China). The HCC cells were transfected with plasmids by ViaFect Transfection Reagent (Promega, WI, USA).

Quantitative real-time PCR (RT-qPCR)

Tissue samples were flash frozen in liquid nitrogen and then lysed with 1 ml RNAiso Plus reagent (Takara Bio, Shiga, Japan). Cells were directly lysed with 1 ml TRIzol. Total RNA was extracted using phenol chloroform and reverse transcribed into cDNA using a PrimeScript™ RT reagent kit (Takara Bio) according to the manufacturer's protocols. The RT-qPCR conditions consisted of 45 cycles, with 15 seconds of denaturation at 95°C and 45 seconds of annealing at 60°C. The reactions were performed in triplicate. The $2^{-\Delta\Delta Cq}$ method was used to calculate the expression of PRL-3 mRNA relative to GAPDH.

Protein extraction and Western blot

The Cell lysates from the total protein extraction for cells were harvested using RIPA buffer (CWbio, Beijing, China). The protein concentration was determined by BCA assays (CWbio, Beijing, China). Nuclear protein extracts were performed with Nuclear and Cytoplasmic Extraction Kit (CWbio, Beijing, China) according to the manufacturer's protocols. The samples were separated by SDS-PAGE and electrotransferred onto 0.22-μm polyvinylidene difluoride

membranes (EMD Millipore, Billerica, MA, USA). The membrane was blocked in 5% fat-free milk and incubated with primary antibodies overnight at 4°C. After being washing with 0.1% TBST, the membranes were incubated with HRP-conjugated secondary antibody. The membranes were further washed with 0.1% TBST and the protein signal on membranes was detected by using an enhanced chemiluminescence system (EMD Millipore, Billerica, MA, USA). The results were quantified using the image analysis tool ImageJ (National Institutes of Health, Bethesda, MD, USA). Each experiment was repeated three times. Each target protein was quantified relative to corresponding GAPDH protein levels.

MTT proliferation assay

Cell proliferation was evaluated by the MTT assay according to the manufacturer's instruction. Briefly, cells were seeded at the density of 1×10^3 cells/well in triplicate into a 96-well plate and incubated for an additional 24 - 96 hours. After incubation for a specific period of time, 20 μL of MTT solution (5 mg/mL; Sigma-Aldrich Co., St Louis, MO, USA) was added to each well and incubated with the cells for 4 hours. Then, 150 μL of dimethyl sulphoxide (DMSO; Sigma-Aldrich Co.) was added to each well, and the cells were incubated for another 15 minutes. The absorbance was read at 490 nm using a microplate reader.

Colony formation

Cells were collected and seeded in 6-well plates at a density of 2.0 × 103 per well, and then incubated for 14 days. Colonies were fixed with methanol, stained with 0.1% crystal violet and counted.

Migration assay

500 μl DMEM supplemented with 10% fetal bovine serum was added to the lower chamber of 24

– well plates. After the transwell culture upper chambers being placed onto 24-well plates, the cells were resuspended in serum free medium (8×10^5 cells/mL for Huh7, Hep3B and HepG2 cells, 4×10^5 cells/mL for Sk-hep-1 cells), and the 100 μL of the suspension was seeded into the upper chamber. After 24 hours, the cells on the lower surface of the upper chambers were fixed in 4% paraformaldehyde, and further stained with 0.25% crystal violet for 20 minutes for microscopic observation of five random fields (100×). The cell migration was evaluated by calculating the number of stained cells. Each experiment was independently repeated in triplicate.

Microarray analysis

PRL-3-overexpressing HCC cells and control cells were used for gene expression profiling. Total RNA extracted from three independent cultures of these two group were used for microarray analysis. Sample labeling and array hybridization were performed according to the Agilent One-Color Microarray-Based Gene Expression Analysis protocol (Agilent Technology). After being washed and fixed, the hybridized arrays were scanned on the Agilent DNA Microarray Scanner (part number G2305C). Microarray analysis was performed by KangChen Bio-tech (Shanghai, China).

Quantification of secreted TGFB1 by ELISA

After addition of 1× protease inhibitor cocktail (CWbio, Beijing, China), total secreted TGFB1 in cell culture supernatant was activated by acidification with 1N HCL and subsequent neutralisation with 1.2N NaOH/0.5M HEPES. The concentration of TGFB1 in each sample was determined using TGF-β ELISA kit (Proteintech Group, Inc; USA) according to the manufacturer's instructions.

Immunofluorescence

For immunofluorescent staining, cells were seeded on observation dish for confocal microscope for 24 hours, fixed with 4% paraformaldehyde for 20 minutes and permeabilized in 0.3% triton X-100 (Solarbio, Beijing, China). for 10 min at room temperature. After being blocked 2% BSA for one hour, the cells were subsequently incubated with primary antibody at 4°C overnight, followed by incubation with immunofluorescent secondary antibody for one hour at room temperature. The nucleus of the fixed cell was stained by DAPI (Solarbio, Beijing, China). Imaging was performed with confocal microscopy. The primary antibodies and immunofluorescent secondary antibodies were showed as followed: Rabbit phospho-FAK (1: 200, ab38512, Abcam); ITGB1(1: 250, Ab24693, Abcam); TGFBR1(1: 100, a0708, Abclonal); Rabbit anti-human C-jun (1: 300, #9165, CST); Rabbit anti-human C-fos (1: 300, ab214672, Abcam); Goat anti-rabbit Alexa Fluor® 647 (1: 800, ab150079, Abcam). Goat anti-rabbit Alexa Fluor® 488 (1: 800, ab150077, Abcam)
